# CMR‐derived left ventricular pressure‐volume loops enhance individualized assessment of disease severity and prognosis in pulmonary arterial hypertension in adults

**DOI:** 10.14814/phy2.70935

**Published:** 2026-05-31

**Authors:** Alessandro Castiglione, Elsa Bergström, Barbro Kjellström, Göran Rådegran, Håkan Arheden, Ellen Ostenfeld

**Affiliations:** ^1^ Clinical Physiology, Department of Clinical Sciences Lund Lund University Lund Sweden; ^2^ Department of Clinical Physiology Skåne University Hospital Lund Sweden; ^3^ Cardiology, Department of Clinical Sciences Lund, Lund University, and Section for Heart Failure and Valvular Disease Skåne University Hospital Lund Sweden

**Keywords:** hemodynamics, left ventricular mechanics, lung transplantation, mortality, pressure‐volume loops, pulmonary arterial hypertension

## Abstract

In pulmonary arterial hypertension (PAH), increased pulmonary vascular resistance (PVR) causes right ventricular pressure overload and left ventricular (LV) underfilling. We investigated LV mechanics and their prognostic value in PAH using cardiovascular magnetic resonance (CMR)‐derived LV pressure‐volume (PV) loops. We included 96 patients (median age 64 years, 70% women) with PAH and clinically indicated CMR between 2003 and 2025, and 32 age‐ and sex‐matched healthy controls. CMR‐based LV PV‐loops were computed. Right heart catheterization, brachial blood pressure, treatment, and time of death or lung transplantation were retrieved from medical records. Patients demonstrated lower stroke work (SW) and ventricular efficiency (VE), and higher contractility (Ees), arterial elastance (Ea), ventriculo‐arterial coupling (VAC), and energy per ejected volume (EEV) than controls. SW and Ea correlated with PVR, VE and VAC correlated with diastolic transpulmonary gradient (*p* < 0.001). After 3 years median follow‐up, 64 (68%) patients died or underwent lung transplantation. Supra‐median SW was associated with better prognosis in patients ≤55 years. Exploratory data suggested increasing SW in treatment responders only. CMR‐based PV‐loops showed LV mechanics alterations in PAH. SW, VE, Ea, and VAC correlated with disease severity. SW predicted survival in patients ≤55 years and might help monitor treatment response.

## 1 INTRODUCTION

Pulmonary arterial hypertension (PAH) is a rare and severe condition characterized by increased resistance in the pulmonary arteries (Humbert et al., 2022) leading to right ventricular (RV) pressure overload and RV failure. Though PAH primarily affects the RV, left ventricular (LV) mechanics are substantially altered via ventricular interdependence and underfilling (Naeije & Badagliacca, 2017; Sjögren et al., 2021). Septal flattening with leftward displacement, pericardial constraint, and reduced atrial inflow lead to progressive reduction of the LV volumes and mass (Burkett et al., 2020; Naeije & Badagliacca, 2017; Sjögren et al., 2021; Venkateshvaran et al., 2024). These changes induce an alteration in the LV mechanics even in the absence of any direct pressure overload on the left side of the heart (Bergström et al., 2025; Hardegree et al., 2013; Jayasekera et al., 2022; Kishiki et al., 2019).

Pressure‐volume (PV) analysis is a well‐established physiological tool for quantifying ventricular mechanics integrating load, contractility, and chamber energetics within a single cardiac cycle. Indices such as stroke work (SW), potential energy (PE), ventricular efficiency (VE), mean external power (MEP), end‐systolic elastance (Ees), effective arterial elastance (Ea), ventriculo‐arterial coupling (VAC), and energy per ejected volume (EEV) can be calculated (Brener et al., 2022; Burkhoff, 2013; Lankhaar et al., 2009). Foundational work has established Ees as a relatively load‐insensitive measure of contractility, while Ea is an indicator of net afterload and thus facilitates ventriculo‐arterial coupling assessment via Ea/Ees (Sunagawa et al., 1985). However, the impact of these LV indices on outcome in PAH is not fully elucidated.

Recently, a non‐invasive model to derive LV pressure‐volume (PV) loops from cardiovascular magnetic resonance (CMR) was proposed. This model requires brachial blood pressure and time‐resolved volumetric CMR‐derived data (Seemann et al., 2019a). Although models for non‐invasive reconstruction of left ventricular PV‐loops using echocardiographic data had been previously established (Antonini‐Canterin et al., 2013; Chen et al., 2001; Gayat et al., 2011), Seeman et al. were the first to develop a completely non‐invasive PV‐loop model based on CMR‐derived volumes, which profits from the higher accuracy of CMR in measurement of end‐diastolic and end‐systolic left ventricular volumes, compared to echocardiography (Fogel et al., 2022). High feasibility and reproducibility across heart‐failure phenotypes have been demonstrated, as well as validation against high‐fidelity catheter measurements in both porcine animal models and humans (Arvidsson et al., 2023; Edlund et al., 2022; Seemann et al., 2024). Despite the growing interest in the use and development of CMR‐based non‐invasive LV PV‐loops (Liu et al., 2024; Nordlund et al., 2024; Reyes et al., 2025), this method has not been used yet to explore LV mechanics in PAH.

Therefore we aimed to (i) investigate and describe LV mechanics alteration by CMR‐derived, non‐invasive LV PV‐loops in a PAH cohort and compare to healthy controls; (ii) explore associations between LV PV‐loop indices and invasive pulmonary hemodynamics from right heart catheterization (RHC) in a PAH cohort; and (iii) evaluate the prognostic value of LV PV‐loop indices for outcome prediction in PAH.

## 2 MATERIALS AND METHODS

### 2.1 Study design and population

This was a retrospective observational study incorporating elements of cohort and case–control design. A retrospective cohort of adult patients who had been referred to the tertiary center at Skåne University Hospital in Lund for investigation of PAH and who underwent clinically indicated CMR between January 2003 and September 2025 were considered for inclusion. Among those, only patients who received a diagnosis of PAH according to the guidelines of the European Society of Cardiology were included (Galie et al., 2004; Galiè et al., 2016; Humbert et al., 2022).

Inclusion criteria were: (i) age ≥ 18 years; (ii) confirmed PAH by clinical discretion in concordance with guidelines (Galiè et al., 2016; Humbert et al., 2022); (iii) availability of a CMR short axis cine stack; and (iv) availability of a brachial arterial blood pressure measured in conjunction to the CMR scan.

Exclusion criteria included: (i) PAH associated with drugs and toxins, with congenital heart disease, or with portal hypertension; (ii) presence of significant left‐sided heart disease unrelated to PAH; (iii) insufficient CMR image quality; (iv) CMR image acquisition during atrial fibrillation or other tachyarrhythmia.

For comparison, a control group consisting of adult healthy volunteers with CMR between 2003 and 2025 included in previous studies (Bergström et al., 2025; Östenson et al., 2023) were matched for age and sex on the group level. Controls were >18 years old, never‐smokers, had no known cardiovascular, pulmonary, or systemic disease including systemic hypertension, had no cardiovascular treatment, and had no pathological findings on electrocardiogram or CMR.

### 2.2 CMR image acquisition

Cardiac magnetic resonance images were acquired using a 1.5 T scanner (Siemens Aera, Siemens Healthineers, Erlangen, Germany) using standardized Society for Cardiovascular Magnetic Resonance (SCMR) protocols (Schulz‐Menger et al., 2020). A contiguous short‐axis cine stack (balanced steady‐state free precession) from base to apex and standard long‐axis 2‐, 3‐, and 4‐chamber views were acquired during breath‐holds with retrospective gating. Typical image parameters were as follows: spatial resolution 1.45 × 1.45 mm, slice thickness 8 mm with 0 mm gap, temporal resolution <46 ms at rest, flip angle 58°–68°, echo time 1.0–1.6 ms, repetition time <42 ms, in accordance with current clinical standards (Schulz‐Menger et al., 2020).

### 2.3 Image analysis and LV volumes

Image analysis with biventricular endocardial and LV epicardial contours was performed using the software Segment v4.1.0.0 R15011 (Medviso, Lund, Sweden) by trained analysts blinded to clinical data. Papillary muscles and trabeculations were included in the blood pool per SCMR post‐processing recommendations (Fogel et al., 2022). Time‐resolved LV volumes (V(t)) as well as biventricular end‐diastolic and end‐systolic volumes, stroke volume (SV), and ejection fraction (EF) were computed.

Two types of subgroup analysis of PAH with patients were performed. First, patients were divided according to the degree of imaging‐based evidence of RV overload into: (i) Mild RV pressure overload and (ii) Severe RV pressure overload using an ad hoc created score based on the CMR criteria contained in the ECS/ERS three‐strata model for comprehensive risk assessment in PAH (Humbert et al., 2022). Accordingly, each patient was assigned a three‐variable score as per Table 1. A score from 0 to 2 was assigned for each variable. Patients with an overall score ≤3 were assigned to the “mild RV pressure overload” group, and patients with an overall score ≥4 were assigned to the “severe RV pressure overload” group.

**TABLE 1:** RV pressure overload score system.

	0 points	1 point	2 points
RVEF [%]	>54	37–54	<37
RVESVI [mL/m^2^]	<42	42–54	>54
SVI [mL/m^2^]	>40	26–40	<26

Abbreviations: RVEF, right ventricular ejection fraction; RVESVI, BSA‐indexed right ventricular end‐systolic volume; SVI, BSA‐indexed left ventricular stroke volume.

As a second subgroup analysis, baseline characteristics and findings were analyzed dichotomizing patients according to age at index CMR examination. Based on previous literature describing differences in PAH pathophysiology and prognosis between older and younger patients, we established an age cut‐off of 55 years to define the two age groups, aiming to create a simplified dichotomic clustering (Hjalmarsson et al., 2018; Rose et al., 2016).

### 2.4 Computation of non‐invasive LV pressure–volume loops

We reconstructed single‐beat LV pressure‐volume loops using a previously published and validated algorithm (Arvidsson et al., 2023; Seemann et al., 2019a, 2024). In brief, a normalized time‐varying elastance curve was scaled to each subject's brachial blood pressures and temporally aligned to cine‐derived LV volumes. The model assumes a volume‐axis intercept of the end‐systolic pressure–volume relationship (V_0_) at zero. The LV peak pressure was approximated from the brachial blood pressure as follows (Seemann et al., 2019a; Westerhof et al., 2005):

LVP=0.83SBP+0.15DBP

where LVP is LV peak pressure at systole, SBP is systolic blood pressure, and DBP is diastolic blood pressure.

The end‐diastolic pressure was estimated as within normal range in all the analyzed participants and set at 7.5 mmHg. This was considered an adequate approximation since non‐PAH related LV dysfunction and post‐capillary pulmonary hypertension had been ruled out in each examined individual. A sensitivity analysis on the effect of different LVEDP values on the used PV‐loop model was previously published and demonstrated minimal impact on the PV‐loop indices used for this study (Seemann et al., 2019a).

**FIGURE 1:**
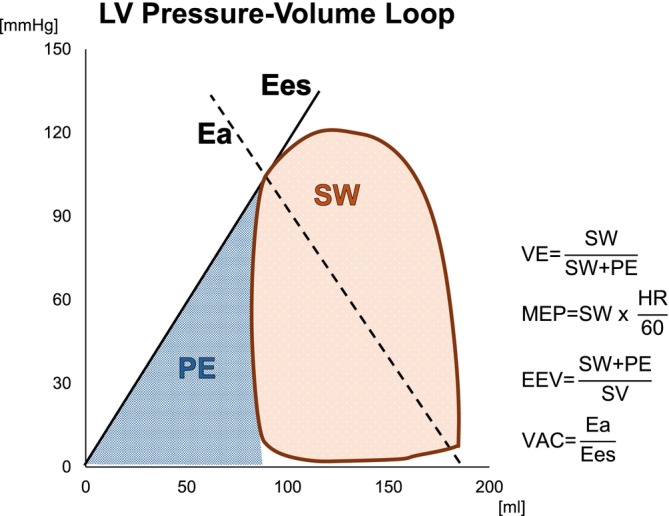
Example of CMR‐based, non‐invasive PV‐loop from a healthy control. The area within the PV‐loop (orange) represents stroke work (SW), the blue area represents the potential energy (PE), the solid straight line represents the end‐systolic elastance (Ees), used as estimation of LV contractility, and the dashed straight line represents the arterial elastance (Ea), as an estimation of the ventricular afterload. On the right side, the formulas used to calculate ventricular efficiency (VE), mean external power (MEP), energy per ejected volume (EEV), and ventriculo‐arterial coupling (VAC). HR, Heart rate; SV, Stroke volume.

From the reconstructed PV‐loops the following was derived (Figure 1): SW (area within the PV‐loop), PE (triangular area between the end‐systolic PV relationship and PV‐loop at end‐systole), mechanical ventricular efficiency (VE = SW/(SW + PE)), mean external power (MEP = SW × heart rate), contractility (Ees = slope of end‐systolic PV relationship), arterial elastance (Ea ≈ LVP/SV), ventriculo‐arterial coupling (VAC = Ea/Ees), and energy per ejected volume (EEV = SW/SV) (Asanoi et al., 1989; Borlaug et al., 2007; Ishihara et al., 1994; Östenson et al., 2023; Sunagawa et al., 1985).

### 2.5 Right‐heart catheterization

Clinically indicated RHC was used for correlation with PV‐loop derived indices if the following conditions were met: (i) RHC had been performed within ±28 days from the CMR; (ii) no changes in PH‐specific treatment had been made between RHC and CMR. Hemodynamic pressures including systolic, diastolic, and mean pulmonary artery pressure (sPAP, dPAP, and mPAP, respectively), and PAWP were acquired during free breathing and averaged over several cardiac cycles. Cardiac output was determined using the thermodilution method. Derived indices included pulmonary vascular resistance (PVR) = (mPAP—PAWP)/cardiac output expressed in Wood units (WU), transpulmonary gradient as mPAP—PAWP, and diastolic transpulmonary gradient as diastolic PAP—PAWP. Healthy controls did not have clinical indications for right heart catheterization.

For the longitudinal investigation, a clinically meaningful improvement at follow‐up was defined as a reduction of both mPAP and PVR values by at least 20% without any reduction in the invasively measured stroke volume.

### 2.6 Outcomes

The endpoint was time from index CMR to first‐coming event of lung transplantation or all‐cause mortality with patients censored at last clinical contact. Time of lung transplantation or death was ascertained from hospital records. End of follow‐up was 30th November 2025.

### 2.7 Statistical analysis

Continuous variables are presented as mean ± standard deviation (SD) or median with interquartile range [IQR]. Comparison between the PAH and control groups or between different PAH subgroups used Student's *t*‐test or Mann–Whitney *U* test as appropriate to Gaussian distribution. Data were assessed for normality using histograms. Categorical variables are presented as absolute number and proportion (%) and compared using the *χ* ^2^ test.

Associations between PV‐loop indices and invasive hemodynamics were assessed using Spearman correlation. Correlations were deemed very strong if the absolute value of the rho coefficient was >0.9, strong for rho values >0.7 and ≤0.9, moderate for rho values >0.5 and ≤0.7, weak for rho values ≤0.5 and >0.3 (Asuero et al., 2006).

Survival was analyzed using Cox proportional hazards models for the PV‐loop indices. Kaplan–Meier curves were plotted with log‐rank tests for survival analysis. First available CMR scan was used for survival analysis and for comparisons with healthy controls, repeat CMR scans were not included.

For patients with repeat CMR scans, first and last available examinations were selected for comparison and changes over time were compared using the Wilcoxon signed‐rank test. In case of repeat CMR scans, only the first one with available RHC data was included for correlation analysis between PV‐loop indices and invasive hemodynamics.

Two‐sided *p* <0.05 were considered statistically significant. Statistical analyses were conducted in SPSS version 29.0 (IBM, Armonk, NY, USA).

## 3 RESULTS

### 3.1 Baseline characteristics

**FIGURE 2:**
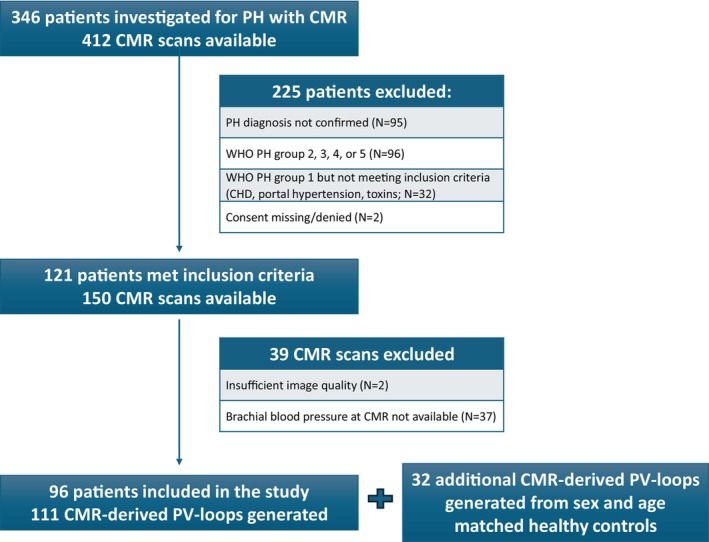
Flow‐chart describing selection process used to include patients and healthy controls and to select the CMR scans for the present study.

Between January 2003 and September 2025, 346 patients with either confirmed or suspected pulmonary hypertension underwent a CMR, whereof 121 patients with a total of 150 CMR scans met the inclusion criteria (Figure 2). Thirty‐nine scans were excluded owing to no systemic arterial blood pressure measurement in conjunction to the CMR scan (*n* = 37) or inadequate image quality (*n* = 2). This left 96 patients and 111 CMR scans for analyses (1 CMR scan = 84 patients, 2 CMR scans = 10 patients and >2 CMR scans = 2 patients). Overall, 111 CMR‐derived non‐invasive PV‐loops could be generated (Figure 2).

At time of baseline CMR examination patients were 64 [45–72] years old and 70% were female (Table 2). Etiology of idiopathic/hereditary and associated PAH (APAH) was evenly distributed; 68% of patients were incident and 55% were treatment‐naïve at baseline. Invasive RHC data at the time‐point of PAH first diagnosis was available for 87 of 96 patients confirming the diagnosis with mPAP of 47 ± 13 mmHg, PAWP of 8 ± 3 mmHg, and PVR of 10.0 ± 5.0 WU. RHC data was available for 63 patients at first CMR examination. Healthy controls did not differ from patients in age, sex or BSA.

**TABLE 2:** Baseline participant characteristics.

	Controls (*n* = 32)	PAH baseline (*n* = 96)	Mild RV pressure overload (*n* = 39)	Severe RV pressure overload (*n* = 57)	*p* value mild vs. severe
Clinical					
Age [y]	61.5 [59–64]	63.5 [45–72]	66 [55–72]	58 [34–71]	0.180
Female	22 (69%)	67 (70%)	32 (82%)	35 (61%)	**0.031**
BMI [kg/m^2^]	24.6 ± 2.7	25.5 ± 4.8	25.5 ± 4.8	25.4 ± 4.8	0.743
BSA [m^2^]	1.85 ± 0.19	1.81 ± 0.23	1.81 ± 0.23	1.83 ± 0.25	0.315
PAH diagnosis group					
IPAH/HPAH	—	50 (52%)	11 (28%)	39 (68%)	**< 0.001**
APAH	—	46 (48%)	28 (72%)	18 (32%)	
Prevalent case	—	30 (32%)	21 (54%)	9 (16%)	**< 0.001**
Incident case	—	66 (68%)	18 (46%)	48 (84%)	
PAH treatment at CMR					
No treatment	—	53 (55%)	18 (46%)	35 (61%)	0.089
Monotherapy	—	21 (22%)	11 (28%)	10 (18%)	
Dual therapy	—	15 (16%)	9 (23%)	6 (11%)	
Triple therapy	—	7 (7%)	1 (3%)	6 (11%)	
Comorbidities					
Diabetes	—	14 (15%)	4 (10%)	10 (18%)	0.320
COPD	—	17 (18%)	5 (13%)	12 (21%)	0.299
Raynaud's disease	—	36 (38%)	22 (56%)	14 (25%)	**0.002**
Ischemic heart disease	—	12 (13%)	5 (13%)	7 (12%)	0.937
Arterial hypertension	—	33 (34%)	13 (33%)	20 (35%)	0.859
Thyroid disease	—	15 (16%)	7 (18%)	8 (14%)	0.604
Atrial fibrillation	—	13 (14%)	3 (8%)	10 (18%)	0.166
Stroke	—	5 (5%)	1 (3%)	4 (7%)	0.335
Dyslipidaemia	—	9 (9%)	4 (10%)	5 (9%)	0.806
Right heart catheterization (*n* = 63)					
mPAP [mmHg]	—	47 ± 13^a^	36 ± 6^b^	53 ± 12^c^	**< 0.001**
dPAP [mmHg]	—	30 ± 10^a^	21 ± 5^b^	34 ± 9^c^	**< 0.001**
sPAP [mmHg]	—	74 ± 20^a^	57 ± 11^b^	83 ± 18^c^	**< 0.001**
PAWP [mmHg]	—	8 ± 4^a^	8 ± 4^b^	8 ± 4^c^	0.430
TPG [mmHg]	—	39 ± 13^a^	28 ± 7^b^	44 ± 12^c^	**< 0.001**
DPG [mmHg]	—	22 ± 10^a^	13 ± 6^b^	26 ± 9^c^	**< 0.001**
PVR [WU]	—	9.4 ± 4.6^a^	5.4 ± 1.9^b^	11.3 ± 4.3^c^	**< 0.001**
SVR [WU]	—	21.2 ± 8.0^a^	17.3 ± 5.1^b^	23.3 ± 8.2^c^	**0.003**
mRAP [mmHg]	—	8 ± 6^a^	4 ± 4^b^	10 ± 5^c^	**< 0.001**
RVEDP [mmHg]	—	12 ± 7^a^	7 ± 4^b^	14 ± 7^c^	**< 0.001**
RVSP [mmHg]	—	72 ± 21^a^	55 ± 14^b^	81 ± 18^c^	**< 0.001**

*Note*: Data expressed as mean ± SD or median [IQR]. Patient characteristics are reported for the whole cohort and for both RV pressure overload categories subsets. Mann–Whitney *U* test, Student's *t*‐test, and *χ* ^2^ test were used to test for differences as appropriate. In the last column to the right, the *p* values refer to comparison of patients with mild vs. severe RV pressure overload. *p* < 0.05 are highlighted in bold. *N* numbers diverging from the ones reported on the top of the table are indicated as follows: ^a^ *n* = 63, ^b^ *n* = 20, and ^c^ *n* = 43.

Abbreviations: APAH, associated pulmonary arterial hypertension; BMI, body mass index; BSA, body surface area; COPD, chronic obstructive pulmonary disease; dPAP, diastolic pulmonary arterial pressure; DPG, diastolic transpulmonary gradient; IPAH/HPAH, idiopathic or hereditary pulmonary arterial hypertension; mPAP, mean pulmonary arterial pressure; mRAP, mean right atrial pressure; PAH, pulmonary arterial hypertension; PAWP, pulmonary arterial wedge pressure; PVR, pulmonary vascular resistance; RVEDP, right ventricular end‐diastolic pressure; RVSP, right ventricular systolic pressure; sPAP, systolic pulmonary arterial pressure; SVR, systemic vascular resistance; TPG, transpulmonary gradient.

Thirty‐nine patients (41%) were assigned as mild RV pressure overload (three‐variables score ≤3) and 57 patients (59%) as severe RV pressure overload (score of ≥4) (Table 2). Patients with mild signs of RV pressure overload were more frequently women and diagnosed with APAH, had lower mPAP and PVR, less signs of pressure overload on CMR, as well as a higher proportion with Raynaud's disease compared to those with severe RV pressure overload.

Stratifying by age category, patients ≤55 years were more likely to have idiopathic or hereditary PAH, be treatment‐naïve, and have higher mPAP and PVR at CMR than patients >55 years, while the proportion of incident cases was alike (69%) in both age groups (Table S1).

### 3.2 Comparison between PAH and controls

Patients with PAH had lower LV volumes, stroke volume, EF, and mass than healthy controls (Table 3). RV volumes were markedly higher in PAH, whereas RV stroke volume and EF were lower than in controls. In the severe RV pressure overload group, end‐diastolic LV volumes and LVEF were lower, and RV volumes were higher compared to the mild overloaded RV group.

**TABLE 3:** CMR and PV‐loop findings.

	Controls (*n* = 32)	Whole PAH cohort (*n* = 96)	Mild RV pressure overload (*n* = 39)	Severe RV pressure overload (*n* = 57)	*p* value mild vs. severe
Clinical					
Non‐invasive blood pressure					
Systolic [mmHg]	121 ± 15	123 ± 18	126 ± 16	120 ± 18	0.068
Diastolic [mmHg]	75 ± 11	76 ± 13	72 ± 11	78 ± 13	**0.040**
Heart rate [bpm]	65 ± 11	79 ± 14***	73 ± 10*	84 ± 14***	**< 0.001**
CMR					
LVEDV [mL]	151 ± 27	130 ± 41**	144 ± 42	121 ± 37***	**0.009**
LVEDVI [mL/m^2^]	81 ± 10	72 ± 19***	80 ± 19	66 ± 17***	**0.001**
LVESV [mL]	61 ± 13	62 ± 24	61 ± 25	62 ± 23	0.839
LVESVI [mL/m^2^]	33 ± 5	34 ± 11	34 ± 12	34 ± 11	0.936
LVSV [mL]	91 ± 16	69 ± 21***	83 ± 19*	59 ± 17***	**< 0.001**
LVSVI [mL/m^2^]	49 ± 6	38 ± 11***	46 ± 8*	33 ± 9***	**< 0.001**
LVEF [%]	60 ± 3	53 ± 8***	59 ± 5	50 ± 7***	**< 0.001**
LVM [g]	74 ± 18	67 ± 22	71 ± 23	64 ± 21*	0.113
LVMI [g/m^2^]	40 ± 7	36 ± 10*	39 ± 10	34 ± 8**	**0.013**
CO [L/min]	5.9 ± 1.1	5.3 ± 1.7*	5.9 ± 1.6	4.9 ± 1.6**	**0.006**
CI [L/min/m^2^]	3.2 ± 0.45	2.9 ± 0.8	3.3 ± 0.8	2.7 ± 0.8**	**0.001**
RVEDV [mL]	156 ± 30	239 ± 77***	188 ± 53**	271 ± 73***	**< 0.001**
RVEDVI [mL/m^2^]	84 ± 12	131 ± 37***	105 ± 23***	148 ± 33***	**< 0.001**
RVESV [mL]	65 ± 15	160 ± 73***	102 ± 35***	200 ± 64***	**< 0.001**
RVESVI [mL/m^2^]	35 ± 6	88 ± 36***	57 ± 16***	109 ± 31***	**< 0.001**
RVSV [mL]	91 ± 16	77 ± 20***	86 ± 22	71 ± 17***	**< 0.001**
RVSVI [mL/m^2^]	49 ± 7	43 ± 10***	48 ± 9	39 ± 9***	**< 0.001**
RVEF [%]	59 ± 3	35 ± 11***	46 ± 6***	27 ± 7***	**< 0.001**
PV‐loop indices					
SW [J]	1.1 ± 0.3	0.9 ± 0.3***	1.1 ± 0.3	0.7 ± 0.3***	**< 0.001**
PE [J]	0.4 ± 0.1	0.4 ± 0.2	0.4 ± 0.2	0.4 ± 0.2	0.456
VE	76 ± 3	68 ± 9***	74 ± 5	64 ± 8***	**< 0.001**
MEP [W]	1.3 ± 0.4	1.1 ± 0.4	1.3 ± 0.4	1.0 ± 0.4**	0.002
EEV [mJ/mL]	16 ± 4	18 ± 3**	17 ± 3	19 ± 4***	**0.013**
Ees [mmHg/mL]	1.5 ± 0.3	1.7 ± 0.6**	1.7 ± 0.56*	1.8 ± 0.7**	0.596
Ea [mmHg/mL]	1.1 ± 0.2	1.7 ± 0.6***	1.3 ± 0.3**	1.9 ± 0.56***	**< 0.001**
VAC	0.7 ± 0.1	1.0 ± 0.3***	0.8 ± 0.2	1.1 ± 0.3***	**< 0.001**

*Note*: Data are expressed as mean ± SD. Patient characteristics are reported for the whole cohort and for both RV pressure overload categories subsets. Student's *t*‐test and *χ* ^2^ test were used to test for differences as appropriate. ** *p* < 0.01, and *** *p* < 0.001 compared to healthy controls. In the last column to the right, the *p* values refer to comparison of patients with mild vs. severe RV pressure overload. *p* < 0.05 are highlighted in bold.

Abbreviations: CI, cardiac index; CO, cardiac output; Ea, left ventricular arterial elastance; Ees, left ventricular end‐systolic elastance; EEV, left ventricular energy per ejected volume; LVEDV, left ventricular end‐diastolic volume; LVEDVI, BSA‐indexed left ventricular end‐diastolic volume; LVEF, left ventricular ejection fraction; LVESV, left ventricular end‐systolic volume; LVESVI, BSA‐indexed left ventricular end‐systolic volume; LVM, left ventricular mass; LVMI, BSA‐indexed left ventricular mass; LVSV, left ventricular stroke volume; LVSVI, BSA‐indexed left ventricular stroke volume; MEP, left ventricular mean external power; PE, left ventricular potential energy; RVEDV, right ventricular end‐diastolic volume; RVEDVI, BSA‐indexed right ventricular end‐diastolic volume; RVEF, right ventricular ejection fraction; RVESV, right ventricular end‐systolic volume; RVESVI, BSA‐indexed right ventricular end‐systolic volume; RVSV, right ventricular stroke volume; RVSVI, BSA‐indexed right ventricular stroke volume; SW, left ventricular stroke work; VAC, left ventricular ventriculo‐arterial coupling; VE, left ventricular efficiency.

Hemodynamic indices derived from the CMR‐based PV‐loops demonstrated that SW and VE were lower and EEV, Ees, Ea, and VAC were higher in PAH than in controls (Table 3).

**FIGURE 3:**
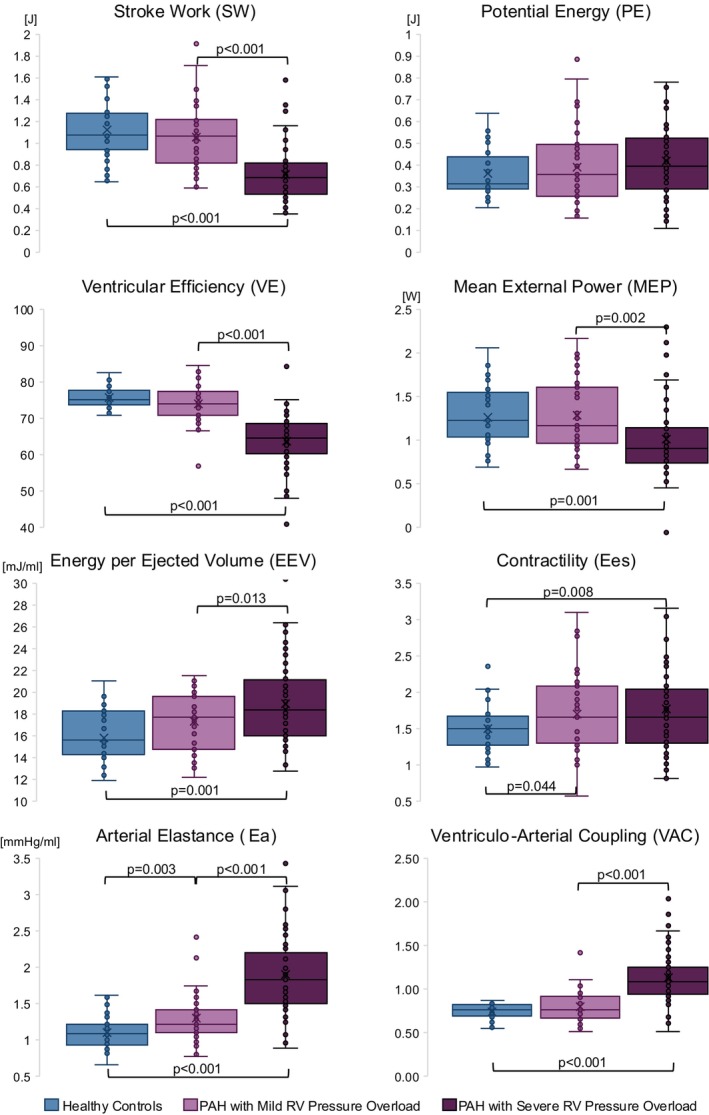
Comparisons of PV‐loop derived hemodynamic indices between healthy controls and patients with PAH. Patients are presented in accordance with the degree of RV pressure overload (mild and severe). Reported *p* values are obtained from unpaired Student's *t* tests between the groups.

Patients with mild RV pressure overload had higher Ees and Ea, while the remaining PV‐loops indices did not differ from the control group. In contrast, patients with severe RV pressure overload exhibited lower SW, VE, and MEP, and higher EEV, Ea, and VAC compared with both controls and patients with mild RV pressure overload. Ees did not differ between the mild and severe RV pressure overload groups (Table 3, Figure 3).

Patients ≤55 years showed a more pronounced RV dilation and RVEF impairment as well as lower VE, lower Ees, and higher VAC compared to patients >55 years (Table S2).

### 3.3 Correlations with invasive measurements

Invasive RHC measurements within 28 days of the CMR scan were available for 67 of the 96 patients with a median elapsed time between CMR and RHC of 1 day [1–2 days].

**FIGURE 4:**
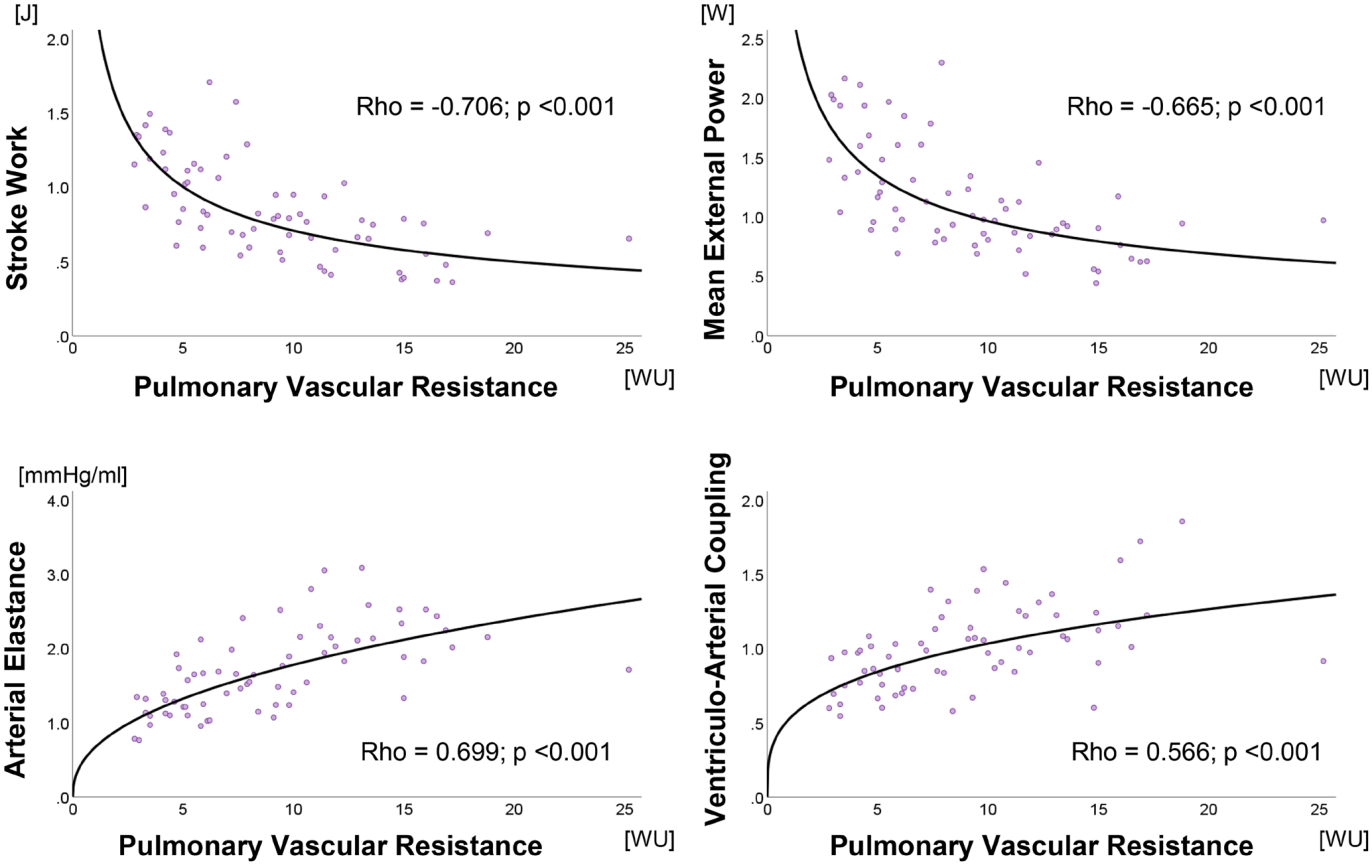
Correlations between pulmonary vascular resistance and stroke work (SW, top left), mean external power (MEP, top right), arterial elastance (Ea, bottom left), and ventriculo‐arterial coupling (VAC, bottom right). *n* = 67.

**FIGURE 5:**
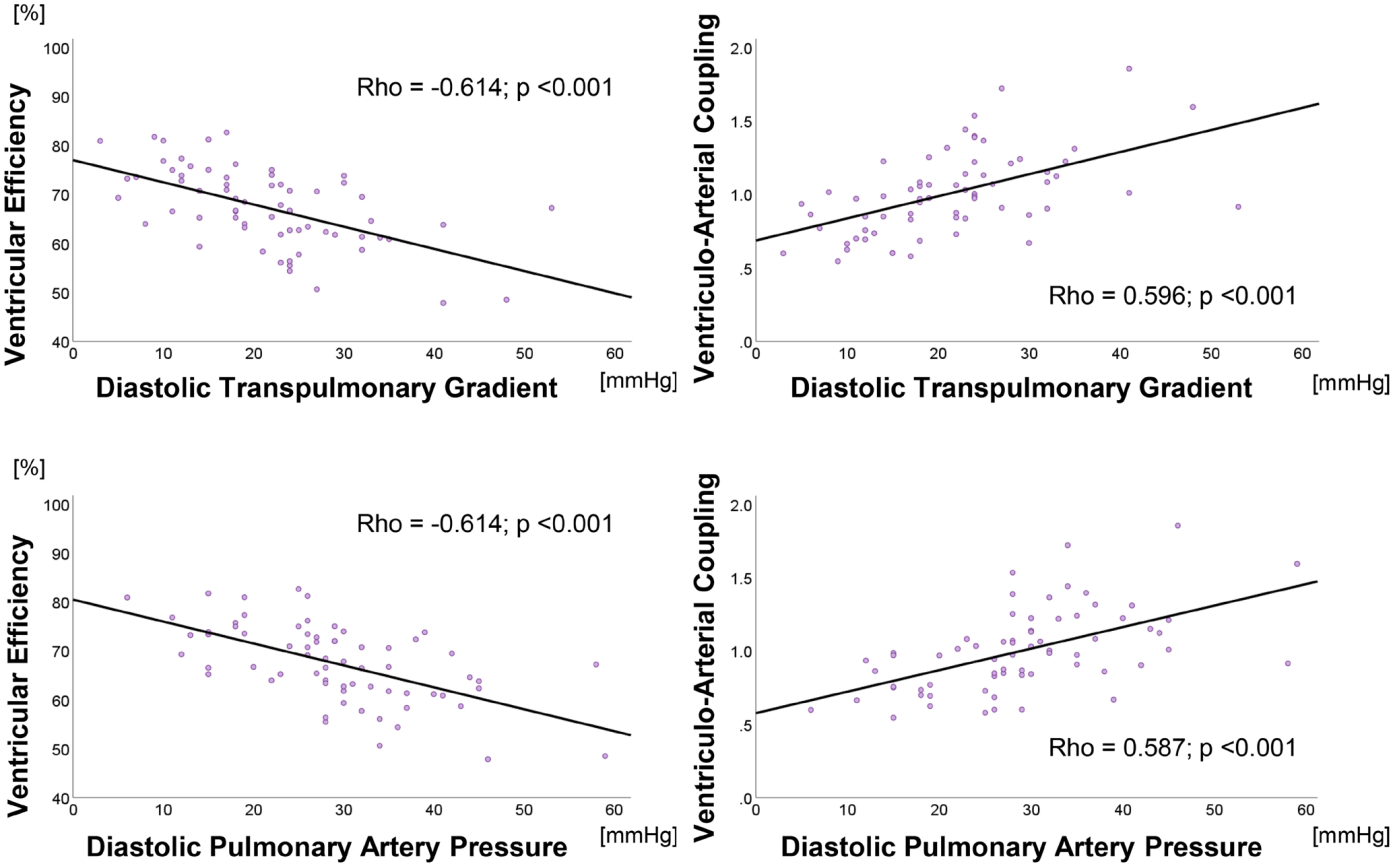
Correlations of diastolic transpulmonary gradient (top row) and diastolic pulmonary arterial pressure (bottom row) versus ventricular efficiency (VE, left) and ventriculo‐arterial coupling (VAC, right). *n* = 67.

VE and VAC correlated moderately with all hemodynamic parameters (Table 4, Figure 4). PVR correlated strongly with SW, and moderately with MEP, Ea, and VAC in a curvilinear fashion (Figure 5). SW, MEP, and Ea correlated weakly to moderately with the remaining hemodynamic parameters. PE and EEV were not found associated with any hemodynamic parameter. (Table 4).

**TABLE 4:** Correlations between PV‐loop and right heart catheterization indices.

*n* = 67	SW	PE	VE	MEP	EEV	Ees	Ea	VAC
mPAP	**−0.468** ***	0.036	**−0.516** ***	**−0.350** **	0.150	0.047	**0.485** ***	**0.516** ***
dPAP	**−0.490** ***	0.085	**−0.614** ***	**−0.344** **	0.133	−0.038	**0.465** ***	**0.587** ***
sPAP	**−0.448** ***	0.044	**−0.493** ***	**−0.351** **	0.151	0.045	**0.462** ***	**0.495** ***
TPG	**−0.491** ***	0.024	**−0.521** ***	**−0.383** **	0.119	0.023	**0.458** ***	**0.526** ***
DPG	**−0.518** ***	0.060	**−0.614** ***	**−0.394** ***	0.091	−0.074	**0.425** ***	**0.596** ***
PVR	**−0.706** ***	−0.105	**−0.576** ***	**−0.665** ***	0.144	0.220	**0.699** ***	**0.566** ***

*Note*: *p* < 0.05 are highlighted in bold.

Abbreviations: dPAP: diastolic pulmonary arterial pressure; DPG: diastolic transpulmonary gradient; Ea: left ventricular arterial elastance; Ees: left ventricular end‐systolic elastance; EEV: left ventricular energy per ejected volume; MEP: left ventricular mean external power; mPAP: mean pulmonary arterial pressure; PE: left ventricular potential energy; PVR: pulmonary vascular resistance; sPAP: systolic pulmonary arterial pressure; SW: left ventricular stroke work; TPG: transpulmonary gradient; VAC: left ventricular ventriculo‐arterial coupling; VE: left ventricular efficiency.

** *p* < 0.01.

*** *p* < 0.001.

### 3.4 Survival analysis

Patients were followed up for a median time of 3.0 [1.3–6.3] years, with one patient lost to follow‐up. Sixty‐five (68%) patients experienced the primary outcome of death (*n* = 53) or lung transplantation (*n* = 12) after a median time of 2.0 [1.0–4.1] years.

Univariate analysis identified age, sex, PAH diagnosis group, and indexed LV mass as associated with the primary endpoint (Table 5). Age‐adjusted bivariate Cox regression confirmed sex as a strong outcome predictor. In the overall PAH population, none of the CMR‐derived ventricular volumes, invasive measures, or LV PV‐loop indices were predictive of survival. RV pressure overload was not associated with outcome.

**TABLE 5:** Uni‐ and bivariate Cox regression analyses.

*n* = 95; outcome = 65	Univariate analysis			Age‐adjusted bivariate analysis		
	HR	95% CI	*p*	HR	95% CI	*p*
Clinical						
Age	1.03	1.01–1.05	**<0.001**	—	—	—
Female Sex	0.45	0.27–0.76	**0.003**	0.40	0.23–0.68	**< 0.001**
APAH diagnosis	1.88	1.14–3.10	**0.013**	1.57	0.95–2.60	0.082
Incident case	0.81	0.48–1.35	0.420	—	—	—
PAH treatment at CMR	0.77	0.47–1.26	0.297	—	—	—
Right heart catheterization (*n* = 63)						
mPAP	1.01	0.99–1.03	0.437	—	—	—
dPAP	1.00	0.97–1.03	0.887	—	—	—
sPAP	1.01	0.99–1.02	0.308	—	—	—
TPG	1.01	0.98–1.04	0.476	—	—	—
DPG	1.00	0.97–1.03	0.980	—	—	—
PVR	1.01	0.94–1.09	0.812	—	—	—
CMR						
LVEDVI	1.00	0.99–1.01	0.973	—	—	—
LVESVI	1.00	0.97–1.02	0.722	—	—	—
LVSVI	1.01	0.98–1.03	0.673	—	—	—
LVEF	1.01	0.98–1.04	0.442	—	—	—
LVMI	1.04	1.01–1.07	**0.016**	1.04	1.01–1.05	**0.012**
CI	1.20	0.91–1.57	0.193	1.32	0.98–1.79	0.069
RVEDVI	1.00	1.00–1.01	0.715	—	—	—
RVESVI	1.00	1.00–1.01	0.853	—	—	—
RVSVI	1.01	0.99–1.04	0.430	—	—	—
RVEF	1.00	0.98–1.02	0.971	—	—	—
Severe RV overload score	1.05	0.64–1.73	0.839	—	—	—
PV‐loop indices						
SW	1.44	0.66–3.16	0.365	—	—	—
PE	1.60	0.34–7.65	0.554	—	—	—
VE	1.01	0.98–1.04	0.614	—	—	—
MEP	1.49	0.86–2.60	0.157	1.68	0.92–3.08	0.091
EEV	0.98	0.91–1.07	0.672	—	—	—
Ees	0.97	0.65–1.46	0.896	—	—	—
Ea	0.82	0.53–1.28	0.383	—	—	—
VAC	0.70	0.31–1.58	0.387	—	—	—

*Note*: Bivariate Cox regression analysis was performed only if the *p* value obtained from the univariate Cox regression analysis was < 0.2. Data expressed as hazard ratios (HR) with 95% confidence intervals (95% CI) for clinical, RHC, CMR, and PV‐loop derived indices. *p* < 0.05 are highlighted in bold.

Abbreviations: APAH, associated pulmonary arterial hypertension; CI, cardiac index; dPAP, diastolic pulmonary arterial pressure; DPG, diastolic transpulmonary gradient; Ea, left ventricular arterial elastance; Ees, left ventricular end‐systolic elastance; EEV, left ventricular energy per ejected volume; LVEDVI, BSA‐indexed left ventricular end‐diastolic volume; LVEF, left ventricular ejection fraction; LVESVI, BSA‐indexed left ventricular end‐systolic volume; LVMI, BSA‐indexed left ventricular mass; LVSVI, BSA‐indexed left ventricular stroke volume; MEP, left ventricular mean external power; mPAP, mean pulmonary arterial pressure; PE, left ventricular potential energy; PVR, pulmonary vascular resistance; RVEDP, right ventricular end‐diastolic pressure; RVEDVI, BSA‐indexed right ventricular end‐diastolic volume; RVEF, right ventricular ejection fraction; RVESVI, BSA‐indexed right ventricular end‐systolic volume; RVSVI, BSA‐indexed right ventricular stroke volume; sPAP, systolic pulmonary arterial pressure; SW, left ventricular stroke work; TPG, transpulmonary gradient; VAC, left ventricular ventriculo‐arterial coupling; VE, left ventricular efficiency.

**FIGURE 6:**
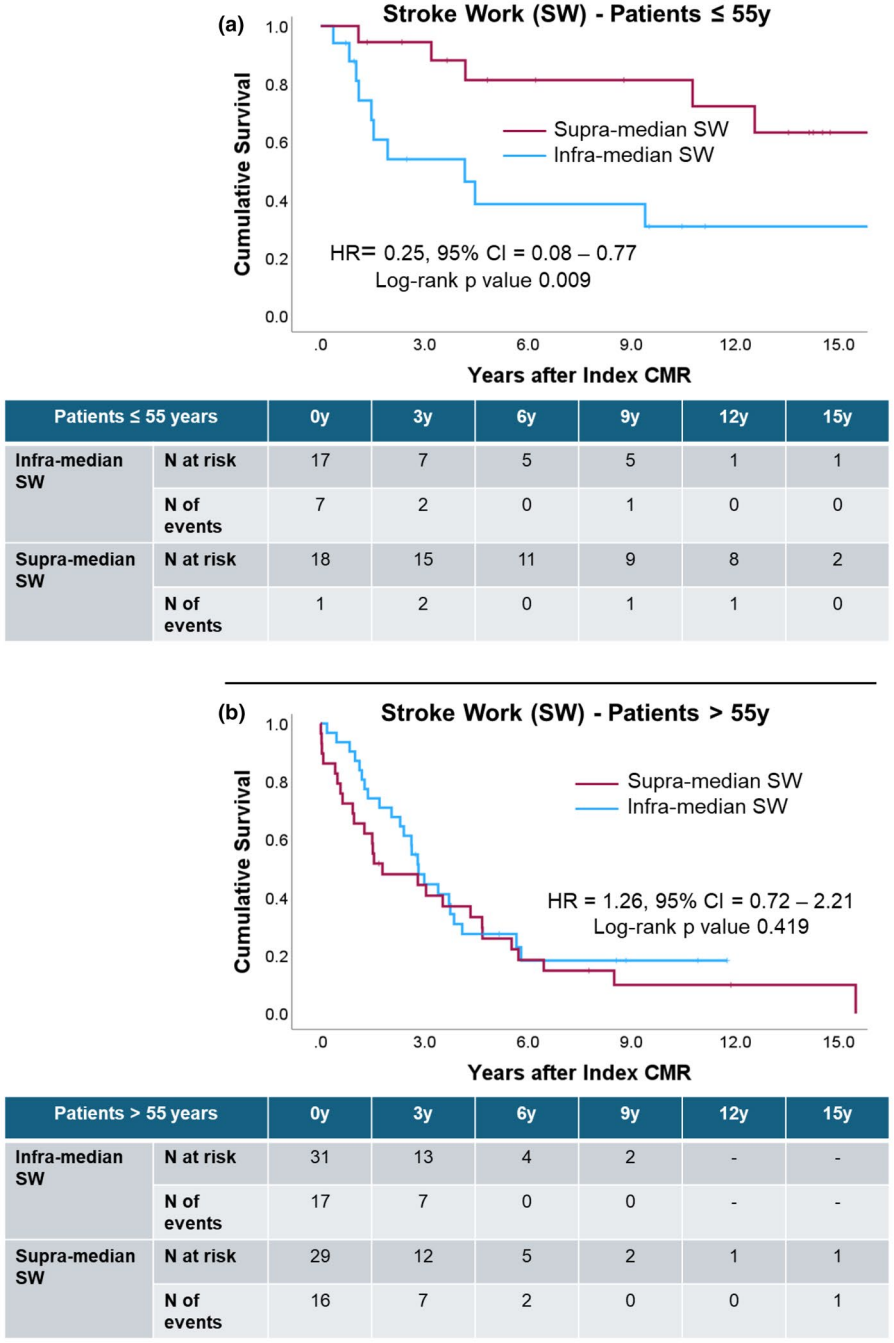
Kaplan–Meier curves and life tables for stroke work: Panel (a) in the subset of patients younger than 55 years at index CMR; panel (b) in the subset of patients older than 55 years at index CMR.

**FIGURE 7:**
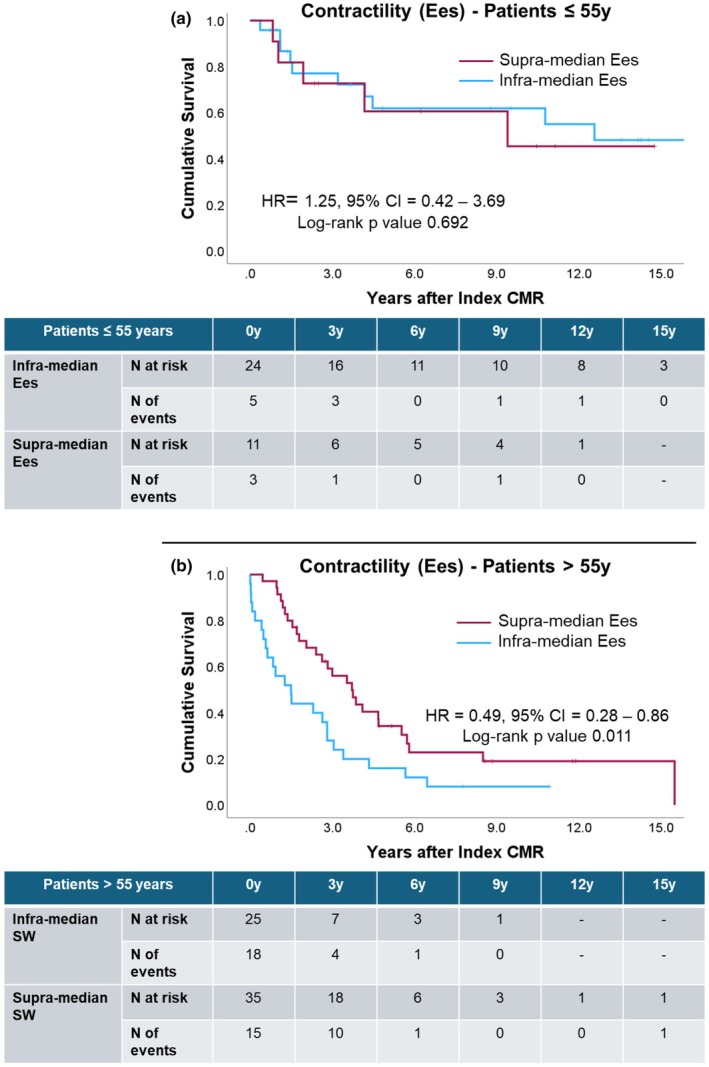
Kaplan–Meier curves and life tables for contractility: Panel (a) in the subset of patients younger than 55 years at index CMR; panel (b) in the subset of patients older than 55 years at index CMR. Figure S1: The graphs show individual changes in SW (above) and MEP (below) from baseline to follow‐up scan in survivors (on the left) and non‐survivors (on the right). Each line represents a single patient and connects the two single values measured in the same individual. Figure S2: The graphs show individual changes in SW (above) and MEP (below) from baseline to follow‐up scan in good‐responders to medical treatment (on the left) and poor‐responders to medical treatment (on the right). Each line represents a single patient and connects the two single values measured in the same individual.

Univariate Cox regression exhibited associations between transplantation‐free survival and sex, indexed LV mass, cardiac output, and PE in patients >55 years (Table 6 and Table 7). Supra‐median SW was associated with longer lung transplantation‐free survival in patients ≤55 years, while in patients >55 years, supra‐median Ees was associated with a more favorable prognosis (Figure 6 and Figure 7).

**TABLE 6:** Univariate Cox regression analysis—Age >55 years.

*n* = 60, outcome = 50	HR	95% CI	*p*
Clinical			
Age	1.02	0.98–1.07	0.290
Female Sex	0.28	0.15–0.52	**<0.001**
APAH diagnosis	1.40	0.78–2.51	0.256
Incident Case	0.67	0.37–1.21	0.184
PAH treatment at CMR	0.94	0.71–1.24	0.661
Right heart catheterization (*n* = 40)			
mPAP	1.02	0.99–1.05	0.253
dPAP	1.02	0.98–1.06	0.365
sPAP	1.01	0.99–1.03	0.236
TPG	1.01	0.98–1.04	0.407
DPG	1.01	0.97–1.06	0.635
PVR	1.03	0.93–1.14	0.602
CMR			
LVEDVI	1.02	1.00–1.03	**0.048**
LVESVI	1.02	1.00–1.05	0.079
LVSVI	1.02	0.99–1.06	0.123
LVEF	0.99	0.95–1.02	0.411
LVMI	1.05	1.02–1.08	**0.002**
CI	1.51	1.03–2.22	**0.035**
RVEDVI	1.01	1.00–1.01	0.096
RVESVI	1.00	1.00–1.01	0.248
RVSVI	1.04	1.01–1.08	**0.031**
RVEF	0.99	0.97–1.02	0.547
Severe RV overload score	1.13	0.65–1.99	0.663
PV‐loop indices			
SW	2.38	0.90–6.29	0.080
PE	11.5	1.99–66.6	**0.006**
VE	0.99	0.95–1.02	0.359
MEP	2.22	1.07–4.63	**0.033**
EEV	1.03	0.94–1.13	0.495
Ees	0.65	0.410–1.04	0.069
Ea	0.72	0.39–1.32	0.292
VAC	1.30	0.50–3.37	0.587

*Note*: Data expressed as hazard ratios (HR) with 95% confidence intervals (95% CI) for clinical, RHC, CMR, and PV‐loop derived indices. *p* < 0.05 are highlighted in bold.

Abbreviations: APAH, associated pulmonary arterial hypertension; CI, cardiac index; CO, cardiac output; dPAP, diastolic pulmonary arterial pressure; DPG, diastolic transpulmonary gradient; Ea, left ventricular arterial elastance; Ees, left ventricular end‐systolic elastance; EEV, left ventricular energy per ejected volume; LVEDVI, BSA‐indexed left ventricular end‐diastolic volume; LVEF, left ventricular ejection fraction; LVESVI, BSA‐indexed left ventricular end‐systolic volume; LVM, left ventricular mass; LVMI, BSA‐indexed left ventricular mass; LVSVI, BSA‐indexed left ventricular stroke volume; MEP, left ventricular mean external power; mPAP, mean pulmonary arterial pressure; PAWP, pulmonary arterial wedge pressure; PE, left ventricular potential energy; PVR, pulmonary vascular resistance; RVEDVI, BSA‐indexed right ventricular end‐diastolic volume; RVEF, right ventricular ejection fraction; RVESVI, BSA‐indexed right ventricular end‐systolic volume; RVSVI, BSA‐indexed right ventricular stroke volume; sPAP, systolic pulmonary arterial pressure; SW, left ventricular stroke work; TPG, transpulmonary gradient; VAC, left ventricular ventriculo‐arterial coupling; VE, left ventricular efficiency.

**TABLE 7:** Univariate Cox regression analysis—Age≤ 55 years.

*n* = 35, outcome = 15	HR	95% CI	*p* value
Clinical			
Age	1.00	0.96–1.05	0.806
Female Sex	0.90	0.25–3.20	0.871
APAH diagnosis	1.86	0.66–5.26	0.241
Incident case	0.82	0.29–2.31	0.702
PAH treatment at CMR	0.93	0.55–1.56	0.779
Right heart catheterization (*n* = 23)			
mPAP	1.04	0.99–1.10	0.086
dPAP	1.04	0.98–1.10	0.221
sPAP	1.03	0.99–1.07	0.157
TPG	1.05	1.00–1.11	0.060
DPG	1.04	0.98–1.11	0.168
PVR	1.14	0.97–1.35	0.116
CMR			
LVEDVI	0.99	0.96–1.01	0.344
LVESVI	0.97	0.92–1.03	0.309
LVSVI	0.98	0.94–1.03	0.439
LVEF	1.00	0.93–1.09	0.933
LVMI	0.96	0.87–1.05	0.320
CI	0.87	0.50–1.51	0.617
RVEDVI	1.01	1.00–1.02	0.323
RVESVI	1.01	1.00–1.02	0.245
RVSVI	0.99	0.94–1.04	0.635
RVEF	0.96	0.91–1.02	0.193
Severe RV overload score	2.00	0.56–7.10	0.283
PV‐loop indices			
SW	0.50	0.10–2.53	0.406
PE	0.17	0.01–11.91	0.327
VE	0.99	0.93–1.06	0.914
MEP	0.67	0.20–2.24	0.514
EEV	0.93	0.79–1.11	0.444
Ees	1.17	0.39–3.50	0.785
Ea	1.06	0.48–2.33	0.892
VAC	1.07	0.19–5.90	0.938

*Note*: Data expressed as hazard ratios (HR) with 95% confidence intervals (95% CI) for clinical, RHC, CMR, and PV‐loop derived indices.

Abbreviations: APAH, associated pulmonary arterial hypertension; CI, cardiac index; dPAP, diastolic pulmonary arterial pressure; DPG, diastolic transpulmonary gradient; Ea, left ventricular arterial elastance; Ees, left ventricular end‐systolic elastance; EEV, left ventricular energy per ejected volume; LVEDVI, BSA‐indexed left ventricular end‐diastolic volume; LVEF, left ventricular ejection fraction; LVESVI, BSA‐indexed left ventricular end‐systolic volume; LVMI, BSA‐indexed left ventricular mass; LVSVI, BSA‐indexed left ventricular stroke volume; MEP, left ventricular mean external power; mPAP, mean pulmonary arterial pressure; PE, left ventricular potential energy; PVR, pulmonary vascular resistance; RVEDP, right ventricular end‐diastolic pressure; RVEDVI, BSA‐indexed right ventricular end‐diastolic volume; RVEF, right ventricular ejection fraction; RVESVI, BSA‐indexed right ventricular end‐systolic volume; RVSVI, BSA‐indexed right ventricular stroke volume; sPAP, systolic pulmonary arterial pressure; SW, left ventricular stroke work; TPG, transpulmonary gradient; VAC, left ventricular ventriculo‐arterial coupling; VE, left ventricular efficiency.

None of the variables LV stroke volume, LV stroke volume BSA‐indexed, systolic systemic arterial pressure, mean systemic arterial pressure, and diastolic systemic arterial pressure were predictive of survival in direct Cox analyses when dichotomizing to subgroups (supra−/infra median levels) in either the whole cohort or in the two different age groups (≤55 and >55 years of age) (Table S3).

### 3.5 Exploratory longitudinal analysis in patients with repeat CMR

In the subset of patients with at least two CMR examinations (*n* = 12; median age 48 years; 67% women), an exploratory descriptive analysis of within‐patient changes over time was performed (Table S4). Median interval between CMR was 15.8 months [5.8–24.7]. More than half had idiopathic or hereditary PAH, and 83% had a change in PAH‐specific treatment between baseline and follow‐up, of which one deescalated treatment. Overall, pulmonary arterial pressures, pressure gradients, and resistance had decreased at follow‐up, though only dPAP, DPG, and PVR decreased significantly.

CMR derived LV volumes, cardiac index as well as RV SV and EF improved from baseline to follow‐up (Table S5). At baseline, RV pressure overload groups were evenly distributed between mild and severe, whereas at follow‐up 58% were classified as severe. There was a trend in increased SW and MEP and decreased Ees and Ea at follow‐up, though only the change in SW reached significance (Table S5). In survivors, SW, PE, and MEP tended to increase, while both indices decreased in patients who died or underwent lung transplantation during follow‐up (Table S6, Figure S1).

PV‐loop changes after treatment initiation or escalation were also assessed. Of nine patients with therapy initiated or intensified after baseline CMR, seven were classified as responders, defined by either a clinically meaningful improvement in invasively measured mPAP and PVR or without major outcome for >5 years. Two patients underwent lung transplantation within 12 months from index CMR without reduction in mPAP or PVR and were classified as non‐responders to medical treatment. Responders were consistently associated with increased SW and MEP, whereas non‐responders were associated with reductions in both indices (Figures S2 and S3).

## 4 DISCUSSION

In a novel approach, the present study showed that LV energetic and ventriculo‐arterial coupling indices could be obtained from CMR‐derived non‐invasive single‐beat LV PV‐loops in a PAH cohort. Compared to healthy controls, patients with mild RV pressure overload showed comparatively preserved LV mechanics whereas in patients with severe RV pressure overload LV PV‐loop indices were altered. LV PV‐loop indices were associated with invasively obtained pulmonary vascular resistance and diastolic pulmonary pressure. In an exploratory survival analysis, age‐stratified LV stroke work and contractility were associated with transplant‐free survival.

These findings support PV‐loops as integrative tools for individualized prognostic assessment and monitoring of treatment response in PAH.

### 4.1 LV PV‐loop phenotype in PAH

The lower LV SW and VE with higher EEV in patients than controls resembles an energetic profile also found in heart failure (Edlund et al., 2022). This supports that LV underfilling is a major driver of these changes, where the LV operates at low volumes with reduced energetic efficiency, implying disproportionate contractile effort for limited preload. This is consistent with prior reports and mechanistic CMR data implicating underfilling rather than primary LV dysfunction in PAH (Guo et al., 2023; Sjögren et al., 2021; Venkateshvaran et al., 2024). Elevated Ees and altered VAC likely reflect a combination of compensatory changes and geometry‐driven effects, aligning with experimental data showing LV hypotrophy in chronic underloading and preserved intrinsic contractile performance when ventricular interdependence is removed (Han et al., 2018; Manders et al., 2014). The interpretation of our data should be careful though. First, LV systolic performance is influenced by pericardially mediated and direct ventricular mechanical interactions in PAH with RV pressure overload, as RV dilatation causes leftward displacement of the interventricular septum, constrained LV filling, and alteration of LV chamber geometry, within a shared intact pericardial space. Furthermore, Ea is inflated by low SV, and single‐beat Ees estimates rise at low LV volumes due to dependence on end‐systolic volume and assumed V_0_ (Bastos et al., 2019; Reyes et al., 2025).

### 4.2 LV hemodynamics and disease severity

Stratifying by RV pressure overload, patients with mild overload had near‐normal LV PV indices, whereas severe overload was associated with marked abnormalities in LV volumes, SW, VE, EEV, Ea, and VAC compared to controls. Ees was similarly elevated in both patient groups; this suggests an early neurohormonal upregulation to preserve systemic pressure despite reduced preload, where the pulmonary vascular disease progresses and RV failure ultimately drive a fall in LV stroke work and efficiency despite persistent high Ees. This is consistent with documented sympathetic activation in PAH where increased LV contractile responsiveness at a given preload despite reduced stroke volume (Han et al., 2018; Rosenkranz et al., 2020). The lack of further Ees increase in patients with severe RV overload may reflect limited adrenergic reserve or intrinsic myocardial changes, for example, fibrosis or impaired relaxation (Broncano et al., 2020).

These findings align with prior invasive PV‐loop research showing limited contractile reserve in mild IPAH and with studies demonstrating that reduced LV longitudinal strain correlated with RV overload and mortality (de Amorim Corrêa et al., 2016; Hardegree et al., 2013; Kasner et al., 2012; Saunders et al., 2018).

### 4.3 Relationship between LV PV‐loop indices and invasive pulmonary hemodynamics

The relationship between LV PV‐loop indices and invasive pulmonary hemodynamics appears physiologically plausible since higher PVR and diastolic pulmonary pressures reflect more advanced pulmonary vascular remodeling and higher RV afterload, worsening RV dysfunction, ventricular interdependence as well as LV underfilling, thereby reducing LV SV and SW. With low SV and preserved systemic pressure, Ea rises while VAC and VE deteriorate (Guo et al., 2023; Sjögren et al., 2021; Venkateshvaran et al., 2024). The strong links between diastolic pulmonary load and VE as well as VAC suggest that small‐vessel disease and vascular stiffness are key determinants of LV energetics, consistent with guideline emphasis on PVR and pulmonary vascular stiffness in precapillary disease severity (Humbert et al., 2022). The ability to detect these associations non‐invasively supports LV PV indices as integrative surrogates of pulmonary vascular load and RV–LV interaction, potentially complementing reliance on invasive assessments.

### 4.4 Prognostic significance of LV structure and hemodynamics

In the present study, age, sex, PAH etiology, and LV mass were associated with survival. When stratified for age below or above 55 years, sex, LV mass, cardiac output, and PE were associated with lung transplant‐free survival. These findings are in line with previous research (Swift et al., 2014, 2017; van Wolferen et al., 2007).

In the full patient cohort, LV PV‐loop indices were not associated with transplant‐free survival, suggesting that these measures may not be suitable for prognosis estimation across the entire heterogeneous PAH population studied. However, exploratory age‐stratified analyses suggested that lower LV stroke work was associated with worse outcomes in patients ≤55 years, whereas lower Ees was associated with worse outcomes in patients >55 years. Also, patients ≤55 years had better survival than the older patient group, despite having a more severe hemodynamic RHC profile, larger RV volumes, similar LV volumes, and worse LV PV‐loop indices (i.e. lower VE, lower Ees, and worse VAC). Beyond the PAH‐related changes, these findings likely reflect effects of lifetime cardiovascular remodeling: aging is associated with LV atrophy and stiffening, increased Ea, and reduced contractile reserve (Chantler & Lakatta, 2012; Chen et al., 1998; Fujimoto et al., 2012). Although Ea and Ees both rise with age, VAC remains relatively constrained (Kass, 2002), implying that older hearts operate at higher baseline contractile demand to match arterial stiffening, leaving limited room for compensations, so efficiency may decline even with similar or milder PAH hemodynamics.

Our findings imply that PV indices may carry age‐dependent prognostic meaning, which may help refine patient risk stratification.

### 4.5 Non‐invasive PV‐loops as a tool for treatment monitoring

In a small longitudinal subset of patients with changes in PAH specific treatment between serial CMR investigations and PV‐loop analysis, treatment responders showed increases in LV SW and MEP, whereas non‐responders had stable or declining values.

Despite the limited sample, this is physiologically plausible: effective therapy reduces PVR and RV afterload, improves RV output, lessens septal shift and pericardial constraint, and increases LV preload and stroke volume, which in turn raises SW and MEP (Aryal et al., 2020; Rosenkranz et al., 2020).

Non‐invasive PV‐loops have been proposed to predict adverse events in heart failure with reduced ejection fraction and as a surrogate endpoint in heart failure (Edlund et al., 2022). Our exploratory findings suggest that this approach may translate to PAH, with PV‐derived SW and MEP serving as integrated, non‐invasive markers of treatment response that reflect RV‐LV interaction and systemic benefit. Larger prospective longitudinal studies with predefined PV‐based response criteria are needed for confirmation.

### 4.6 Clinical implications

Non‐invasive CMR‐derived PV‐loop analysis offers a potentially attractive approach to quantify LV energetic state and coupling in PAH without invasive LV catheterization. This is particularly relevant given the central role of RV‐LV interaction in PAH pathophysiology. While standard CMR metrics already provide valuable information, PV‐loop analysis may complement those, providing a clinically feasible way of quantifying LV energetics and ventriculo‐arterial coupling in a non‐invasive way. At present, the data support PV‐loop indices primarily as physiological descriptors and as correlates of pulmonary hemodynamic severity. Future studies are required to investigate whether these indices offer incremental clinical value to risk‐stratify patients, guide therapy, or track responsiveness to treatment.

### 4.7 Limitations

Some limitations merit consideration. Although the underlying method has been validated against invasive LV pressure in animal and human studies with good agreement for key indices (Seemann et al., 2024; Arvidsson et al., 2022), non‐invasive PV‐loop reconstruction relies on assumptions regarding pressure calibration and elastance waveform shape. Moreover, we used V_0_ = 0, LV end‐diastolic pressure of 7.5 mmHg, and left ventricular peak pressure as LVP = 0.83 SBP + 0.15 DBP in all individuals. While these input measures in the model may be theoretical assumptions, they have been assessed valid in computation of non‐invasive PV‐loop parameters in the absence of increased LV filling pressures (Seemann et al., 2019a; Arvidsson et al., 2023; Seemann et al., 2024; Arvidsson et al., 2022). Brachial blood pressure is an imperfect surrogate for central LV pressure, and model‐based estimation of LV peak pressure and LV end‐diastolic pressure introduces uncertainty. Second, this was a retrospective study spanning a long inclusion period, and differences in imaging protocols, clinical management, and disease phenotype across time may introduce heterogeneity. Third, group stratification by RV pressure overload may be confounded by differences in PAH subtype distribution, treatment status, and comorbidity burden. Also, both our age and RV pressure overload cut‐offs, while clinically reasonable and consistent with previous literature (Dardi et al., 2024; Humbert et al., 2022), remain somewhat arbitrary; future work might explore more granular age categories and risk‐stratified analyses. Further, the CMR PAH database from our tertiary center possibly collects data from a selected patient population and might not reflect the general PAH population. Finally, some analyses were exploratory and involved multiple comparisons; therefore, findings should be interpreted conservatively, especially regarding prognosis.

## 5 CONCLUSIONS

Non‐invasive CMR‐derived LV PV‐loop analysis reveals an LV energetic phenotype in PAH characterized by reduced SW and mechanical efficiency, with the greatest abnormalities seen in patients with imaging‐based evidence of severe RV pressure overload. PV‐loop indices correlate with invasive measures of pulmonary hemodynamic severity, supporting the concept that LV energetic impairment in PAH is closely linked to disease severity and ventricular interdependence. While prognostic associations are explorative and require further investigation, they suggest that LV PV‐loop analysis may provide a valuable physiological framework for understanding LV consequences of PAH and motivates prospective studies assessing reproducibility, sensitivity to change in medical treatment, and incremental clinical value beyond established imaging and hemodynamic markers.

## AUTHOR CONTRIBUTIONS

**Alessandro Castiglione:** Data curation; formal analysis; investigation. **Elsa Bergström:** Data curation. **Barbro Kjellström:** Conceptualization; data curation; supervision. **Göran Rådegran:** Conceptualization; data curation. **Håkan Arheden:** Supervision. **Ellen Ostenfeld:** Conceptualization; investigation; project administration; supervision.

## FUNDING INFORMATION

This study was funded by the Swedish Research Council, Swedish Heart Lung Foundation, Southern Healthcare Region of Sweden, Swedish Society of Medicine, Swedish governmental funding of clinical research (ALF), Skåne University Hospital, Crafoord Foundation, and Lund University. The funding agencies have not had any influence on the design of the study or interpretation of the results.

## CONFLICT OF INTEREST STATEMENT

The authors have no known competing financial interests or personal relationships that could have appeared to influence the work reported in this paper.

## ETHICS STATEMENT

The study was approved by the Ethical Review Board, Lund, Sweden (application numbers 2010/114, 2010/248, 2011/777), and all participants provided written informed consent where required. The study was conducted in accordance with the Helsinki Declaration and reported in accordance with the STROBE recommendations (Rothwell & Bhatia, 2007).

## Supporting information


**Figure S1.** The graphs show individual changes in SW (above) and MEP (below) from baseline to follow‐up scan in survivors (on the left) and non‐survivors (on the right). Each line represents a single patient and connects the two single values measured in the same individual.
**Figure S2.** The graphs show individual changes in SW (above) and MEP (below) from baseline to follow‐up scan in good‐responders to medical treatment (on the left) and poor‐responders to medical treatment (on the right). Each line represents a single patient and connects the two single values measured in the same individual.
**Figure S3.** The image shows two examples of how MR‐based, non‐invasive PV‐loops changed from baseline to follow‐up in a patient who responded well to PAH‐specific medical treatment (above) and in a patient who did not respond to medical treatment (below). The treatment non‐responder shown here underwent lung transplantation three months after follow‐up.

**Table S1.** Age‐Stratified Baseline Participant Characteristics.
**Table S2.** Age‐Stratified Baseline CMR and PV‐loop findings.
**Table S3.** Univariate Cox regression analysis.
**Table S4.** Repeat scans patient characteristics.
**Table S5.** Repeat scans patient CMR and PV‐loop findings.
**Table S6.** PV‐loop indices in repeat scans in non‐survivors and survivors.

## Data Availability

The data underlying this article will be shared upon reasonable request to the corresponding author.

## References

[phy270935-bib-0001] Antonini‐Canterin, F. , Poli, S. , Vriz, O. , Pavan, D. , Bello, V. D. , & Nicolosi, G. L. (2013). The ventricular‐arterial coupling: From basic pathophysiology to clinical application in the echocardiography laboratory. Journal of Cardiovascular Echography, 23(4), 91–95. 10.4103/2211-4122.127408 28465893 PMC5353400

[phy270935-bib-0002] Arvidsson, P. , Green, P. G. , Watson, W. D. , Shanmuganathan, M. , Heiberg, E. , & De Maria, G. L. (2022). Invasive validation of pressure‐volume loops derived from cardiovascular magnetic resonance imaging and brachial blood pressure in heart failure patients. European Heart Journal, 43(Supplement_2), 229. 10.1093/eurheartj/ehac544.229 PMC10631830 37969333

[phy270935-bib-0003] Arvidsson, P. M. , Green, P. G. , Watson, W. D. , Shanmuganathan, M. , Heiberg, E. , De Maria, G. L. , Arheden, H. , Herring, N. , & Rider, O. J. (2023). Non‐invasive left ventricular pressure‐volume loops from cardiovascular magnetic resonance imaging and brachial blood pressure: Validation using pressure catheter measurements. European Heart Journal ‐ Imaging Methods and Practice, 1(2), qyad035. 10.1093/ehjimp/qyad035 37969333 PMC10631830

[phy270935-bib-0004] Aryal, S. R. , Sharifov OF , & Lloyd, S. G. (2020). Emerging role of cardiovascular magnetic resonance imaging in the management of pulmonary hypertension. European Respiratory Review, 29(156), 190138. 10.1183/16000617.0138-2019 32620585 PMC9488921

[phy270935-bib-0005] Asanoi, H. , Sasayama, S. , & Kameyama, T. (1989). Ventriculoarterial coupling in normal and failing heart in humans. Circulation Research, 65(2), 483–493. 10.1161/01.res.65.2.483 2752553

[phy270935-bib-0006] Asuero, A. G. , Sayago, A. , & González, A. (2006). The correlation coefficient: An overview. Critical Reviews in Analytical Chemistry, 36(1), 41–59.

[phy270935-bib-0007] Bastos, M. B. , Burkhoff, D. , Maly, J. , Daemen, J. , den Uil, C. A. , Ameloot, K. , Lenzen, M. , Mahfoud, F. , Zijlstra, F. , Schreuder, J. J. , & Van Mieghem, N. M. (2019). Invasive left ventricle pressure–volume analysis: Overview and practical clinical implications. European Heart Journal, 41(12), 1286–1297. 10.1093/eurheartj/ehz552 PMC7084193 31435675

[phy270935-bib-0008] Bergström, E. , Pola, K. , Kjellström, B. , Töger, J. , Arvidsson, P. , Carlsson, M. , Arvidsson, P. M. , Rådegran, G. , Arheden, H. , & Ostenfeld, E. (2025). Increased contractility affects left ventricular kinetic energy in pulmonary hypertension. Physiological Reports, 13(17), e70563. 40939108 10.14814/phy2.70563 PMC12431581

[phy270935-bib-0009] Borlaug, B. A. , Melenovsky, V. , Redfield, M. M. , Kessler, K. , Chang, H.‐J. , Abraham, T. P. , & Kass, D. A. (2007). Impact of arterial load and loading sequence on left ventricular tissue velocities in humans. Journal of the American College of Cardiology, 50(16), 1570–1577. 17936156 10.1016/j.jacc.2007.07.032

[phy270935-bib-0010] Brener, M. I. , Masoumi, A. , Ng, V. G. , Tello, K. , Bastos, M. B. , Cornwell, I. I. I. W. K. , Cornwell, W. K. , Hsu, S. , Tedford, R. J. , Lurz, P. , Rommel, K. P. , Kresoja, K. P. , Nagueh, S. F. , Kanwar, M. K. , Kapur, N. K. , Hiremath, G. , Sarraf, M. , van den Enden, A. J. M. , van Mieghem, N. M. , … Burkhoff, D. (2022). Invasive right ventricular pressure‐volume analysis: Basic principles, clinical applications, and practical recommendations. Circulation. Heart Failure, 15(1), e009101. 34963308 10.1161/CIRCHEARTFAILURE.121.009101 PMC8766922

[phy270935-bib-0011] Broncano, J. , Bhalla, S. , Gutierrez, F. R. , Vargas, D. , Williamson, E. E. , Makan, M. , & Luna, A. (2020). Cardiac MRI in pulmonary hypertension: From magnet to bedside. Radiographics, 40(4), 982–1002. 10.1148/rg.2020190179 PubMed PMID: 32609599. 32609599

[phy270935-bib-0012] Burkett, D. A. , Patel, S. S. , Mertens, L. , Friedberg, M. K. , & Ivy, D. D. (2020). Relationship between left ventricular geometry and invasive hemodynamics in pediatric pulmonary hypertension. Circulation: Cardiovascular Imaging, 13(5), e009825. 32408829 10.1161/CIRCIMAGING.119.009825 PMC7236425

[phy270935-bib-0013] Burkhoff, D. (2013). Pressure‐volume loops in clinical research: A contemporary view (pp. 1173–1176). American College of Cardiology Foundation. 10.1016/j.jacc.2013.05.049 23770176

[phy270935-bib-0014] Chantler, P. D. , & Lakatta, E. G. (2012). Arterial‐ventricular coupling with aging and disease. Frontiers in Physiology, 3, 90. 10.3389/fphys.2012.00090 22586401 PMC3345942

[phy270935-bib-0015] Chen, C.‐H. , Fetics, B. , Nevo, E. , Rochitte, C. E. , Chiou, K.‐R. , Ding, P.‐A. , Kawaguchi, M. , & Kass, D. A. (2001). Noninvasive single‐beat determination of left ventricular end‐systolic elastance in humans. JACC, 38(7), 2028–2034. 10.1016/S0735-1097(01)01651-5 11738311

[phy270935-bib-0016] Chen, C.‐H. , Nakayama, M. , Nevo, E. , Fetics, B. J. , Maughan, W. L. , & Kass, D. A. (1998). Coupled systolic‐ventricular and vascular stiffening with age: Implications for pressure regulation and cardiac reserve in the elderly. Journal of the American College of Cardiology, 32(5), 1221–1227. 9809929 10.1016/s0735-1097(98)00374-x

[phy270935-bib-0017] Dardi, F. , Boucly, A. , Benza, R. , Frantz, R. , Mercurio, V. , Olschewski, H. , Rådegran, G. , Rubin, L. J. , & Hoeper, M. M. (2024). Risk stratification and treatment goals in pulmonary arterial hypertension. The European Respiratory Journal, 64(4), 2401323. 10.1183/13993003.01323-2024 39209472 PMC11525341

[phy270935-bib-0018] de Amorim Corrêa, R. , de Oliveira, F. B. , Barbosa, M. M. , Barbosa, J. A. A. , Carvalho, T. S. , & Barreto, M. C. (2016). Left ventricular function in patients with pulmonary arterial hypertension: The role of two‐dimensional speckle tracking strain. Echocardiography (Mount Kisco, N.Y.), 33(9), 1326–1334. 10.1111/echo.13267 27460782

[phy270935-bib-0019] Edlund, J. , Arvidsson, P. M. , Nelsson, A. , Smith, J. G. , Magnusson, M. , Heiberg, E. , Steding‐Ehrenborg, K. , & Arheden, H. (2022). Noninvasive assessment of left ventricular pressure‐volume relations: Inter‐and intraobserver variability and assessment across heart failure subtypes. The American Journal of Cardiology, 184, 48–55. 36192197 10.1016/j.amjcard.2022.09.001

[phy270935-bib-0020] Fogel, M. A. , Anwar, S. , Broberg, C. , Browne, L. , Chung, T. , Johnson, T. , Muthurangu, V. , Taylor, M. , Valsangiacomo‐Buechel, E. , & Wilhelm, C. (2022). Society for Cardiovascular Magnetic Resonance/European Society of Cardiovascular Imaging/American Society of Echocardiography/Society for Pediatric Radiology/north American Society for Cardiovascular Imaging Guidelines for the use of cardiovascular magnetic resonance in pediatric congenital and acquired heart disease: Endorsed by the American Heart Association. Journal of Cardiovascular Magnetic Resonance, 24(1), 37. 10.1186/s12968-022-00843-7 35725473 PMC9210755

[phy270935-bib-0021] Fujimoto, N. , Hastings, J. L. , Bhella, P. S. , Shibata, S. , Gandhi, N. K. , & Carrick‐Ranson, G. (2012). Effect of ageing on left ventricular compliance and distensibility in healthy sedentary humans. Journal of Physiology, 590(8), 1871–1880. 10.1113/jphysiol.2011.218271 22331419 PMC3573309

[phy270935-bib-0023] Galie, N. , Torbicki, A. , Barst, R. , Dartevelle, P. , Haworth, S. , Higenbottam, T. , Olschewski, H. , Peacock, A. , Pietra, G. , Rubin, L. J. , & Simonneau, G. (2004). Guidelines on diagnosis and treatment of pulmonary arterial hypertension: the task force on diagnosis and treatment of pulmonary arterial hypertension of the European Society of Cardiology. European Heart Journal, 25(24), 2243–2278. 15589643 10.1016/j.ehj.2004.09.014

[phy270935-bib-0022] Galiè, N. , Humbert, M. , Vachiery, J.‐L. , Gibbs, S. , Lang, I. , Torbicki, A. , Simonneau, G. , Peacock, A. , Vonk Noordegraaf, A. , Beghetti, M. , Ghofrani, A. , Gomez Sanchez, M. A. , Hansmann, G. , Klepetko, W. , Lancellotti, P. , Matucci, M. , McDonagh, T. , Pierard, L. A. , Trindade, P. T. , … ESC Scientific Document Group . (2016). 2015 ESC/ERS guidelines for the diagnosis and treatment of pulmonary hypertension: The joint task force for the diagnosis and treatment of pulmonary hypertension of the European Society of Cardiology (ESC) and the European Respiratory Society (ERS): Endorsed by: Association for European Paediatric and Congenital Cardiology (AEPC), International Society for Heart and Lung Transplantation (ISHLT). European Heart Journal, 37(1), 67–119.26320113

[phy270935-bib-0024] Gayat, E. , Mor‐Avi, V. , Weinert, L. , Yodwut, C. , & Lang, R. M. (2011). Noninvasive quantification of left ventricular elastance and ventricular‐arterial coupling using three‐dimensional echocardiography and arterial tonometry. American Journal of Physiology. Heart and Circulatory Physiology, 301(5), H1916–H1923. 10.1152/ajpheart.00760.2011 21908790

[phy270935-bib-0025] Guo, J. , Wang, J. , Wang, L. , Li, Y. , Xu, Y. , Li, W. , Chen, C. , He, J. , Yin, L. , Pu, S. , & Wen, B. (2023). Left ventricular underfilling in PAH: A potential indicator for adaptive‐to‐maladaptive transition. Pulmonary Circulation, 13(4), e12309. 10.1002/pul2.12309 38045097 PMC10689890

[phy270935-bib-0026] Han, J.‐C. , Guild, S.‐J. , Pham, T. , Nisbet, L. , Tran, K. , Taberner, A. J. , & Loiselle, D. S. (2018). Left‐ventricular energetics in pulmonary arterial hypertension‐induced right‐ventricular hypertrophic failure. Frontiers in Physiology, 8, 1115. 29375394 10.3389/fphys.2017.01115 PMC5767264

[phy270935-bib-0027] Hardegree, E. L. , Sachdev, A. , Fenstad, E. R. , Villarraga, H. R. , Frantz, R. P. , McGoon, M. D. , Oh, J. K. , Ammash, N. M. , Connolly, H. M. , Eidem, B. W. , & Pellikka, P. A. (2013). Impaired left ventricular mechanics in pulmonary arterial hypertension: Identification of a cohort at high risk. Circulation: Heart Failure, 6(4), 748–755. 23709658 10.1161/CIRCHEARTFAILURE.112.000098

[phy270935-bib-0028] Hjalmarsson, C. , Rådegran, G. , Kylhammar, D. , Rundqvist, B. , Multing, J. , Nisell, M. D. , Kjellström, B. , & SveFPH and SPAHR . (2018). Impact of age and comorbidity on risk stratification in idiopathic pulmonary arterial hypertension. The European Respiratory Journal, 51(5), 1702310. 10.1183/13993003.02310-2017 29622568

[phy270935-bib-0029] Humbert, M. , Kovacs, G. , Hoeper, M. M. , Badagliacca, R. , Berger, R. M. , Brida, M. , Carlsen, J. , Coats, A. J. S. , Escribano‐Subias, P. , Ferrari, P. , Ferreira, D. S. , Ghofrani, H. A. , Giannakoulas, G. , Kiely, D. G. , Mayer, E. , Meszaros, G. , Nagavci, B. , Olsson, K. M. , Pepke‐Zaba, J. , … Quint, J. K. (2022). ESC/ERS Guidelines for the diagnosis and treatment of pulmonary hypertension: Developed by the task force for the diagnosis and treatment of pulmonary hypertension of the European Society of Cardiology (ESC) and the European Respiratory Society (ERS). Endorsed by the International Society for Heart and Lung Transplantation (ISHLT) and the European reference network on rare respiratory diseases (ERN‐LUNG). European Heart Journal, 43(38), 3618–3731. 36017548 10.1093/eurheartj/ehac237

[phy270935-bib-0030] Ishihara, H. , Yokota, M. , Sobue, T. , & Saito, H. (1994). Relation between ventriculoarterial coupling and myocardial energetics in patients with idiopathic dilated cardiomyopathy. Journal of the American College of Cardiology, 23(2), 406–416. 10.1016/0735-1097(94)90428-6 8294695

[phy270935-bib-0031] Jayasekera, G. , Macdonald, A. , Mccomb, C. , Orchard, V. , Welsh, D. , Church, C. , Johnson, M. , Brewis, M. , Berry, C. , Radjenovic, A. , & Peacock, A. (2022). Left ventricular dysfunction and intra‐ventricular dyssynchrony in idiopathic pulmonary arterial hypertension. International Journal of Cardiology, 365, 131–139. 35870633 10.1016/j.ijcard.2022.07.032

[phy270935-bib-0032] Kasner, M. , Westermann, D. , Steendijk, P. , Dröse, S. , Poller, W. , Schultheiss, H. P. , & Tschöpe, C. (2012). Left ventricular dysfunction induced by nonsevere idiopathic pulmonary arterial hypertension: a pressure‐volume relationship study. American Journal of Respiratory and Critical Care Medicine, 186(2), 181–189. 10.1164/rccm.201110-1860OC 22561959

[phy270935-bib-0033] Kass, D. A. (2002). Age‐related changes in venticular–arterial coupling: Pathophysiologic implications. Heart Failure Reviews, 7(1), 51–62. 11790922 10.1023/a:1013749806227

[phy270935-bib-0034] Kishiki, K. , Singh, A. , Narang, A. , Gomberg‐Maitland, M. , Goyal, N. , Maffessanti, F. , Besser, S. A. , Mor‐Avi, V. , Lang, R. M. , & Addetia, K. (2019). Impact of severe pulmonary arterial hypertension on the left heart and prognostic implications. Journal of the American Society of Echocardiography, 32(9), 1128–1137. 31278050 10.1016/j.echo.2019.05.008 PMC7147873

[phy270935-bib-0035] Lankhaar, J.‐W. , Rövekamp, F. A. , Steendijk, P. , Faes, T. J. , Westerhof, B. E. , Kind, T. , Faes, T. J. C. , Vonk‐Noordegraaf, A. , & Westerhof, N. (2009). Modeling the instantaneous pressure–volume relation of the left ventricle: A comparison of six models. Annals of Biomedical Engineering, 37(9), 1710–1726. 19554450 10.1007/s10439-009-9742-x PMC3233835

[phy270935-bib-0036] Liu, J. , Bilgi, C. , Bregasi, A. , Mitchell, G. F. , & Pahlevan, N. M. (2024). Noninvasive left ventricle pressure‐volume loop determination method with cardiac magnetic resonance imaging and carotid tonometry using a physics‐informed approach. IEEE Journal of Biomedical and Health Informatics, 28(9), 5487–5496. 10.1109/jbhi.2024.3412671 38861439

[phy270935-bib-0037] Manders, E. , Bogaard, H.‐J. , Handoko, M. L. , Veerdonk, M. C. , Keogh, A. , Westerhof, N. , Stienen, G. J. , Dos Remedios, C. G. , Humbert, M. , Dorfmüller, P. , & Fadel, E. (2014). Contractile dysfunction of left ventricular cardiomyocytes in patients with pulmonary arterial hypertension. JACC, 64(1), 28–37. 10.1016/j.jacc.2014.04.031 24998125

[phy270935-bib-0038] Naeije, R. , & Badagliacca, R. (2017). The overloaded right heart and ventricular interdependence. Cardiovascular Research, 113(12), 1474–1485. 28957537 10.1093/cvr/cvx160

[phy270935-bib-0039] Nordlund, D. , Lav, T. , Jablonowski, R. , Khoshnood, A. , Ekelund, U. , Atar, D. , Erlinge, D. , Engblom, H. , & Arheden, H. (2024). Contractility, ventriculoarterial coupling, and stroke work after acute myocardial infarction using CMR‐derived pressure‐volume loop data. Clinical Cardiology, 47(1), e24216. 10.1002/clc.24216 38269628 PMC10790509

[phy270935-bib-0040] Östenson, B. , Ostenfeld, E. , Edlund, J. , Heiberg, E. , Arheden, H. , & Steding‐Ehrenborg, K. (2023). Endurance‐trained subjects and sedentary controls increase ventricular contractility and efficiency during exercise: Feasibility of hemodynamics assessed by non‐invasive pressure‐volume loops. PLoS One, 18(5), e0285592. 37163493 10.1371/journal.pone.0285592 PMC10171617

[phy270935-bib-0041] Reyes, L. J. , Alfaro, M. E. , Kireta, J. A. , Joyner, M. L. , Ullas, S. , LaViolette, B. , Hirenallur‐Shanthappa, D. , & Tania, N. (2025). Assessment of a non‐invasive approach for pressure volume loop prediction in mice. Scientific Reports, 15(1), 18216. 40414963 10.1038/s41598-025-00691-2 PMC12104428

[phy270935-bib-0042] Rose, J. A. , Cleveland, J. M. , Rao, Y. , Minai, O. A. , & Tonelli, A. R. (2016). Effect of age on phenotype and outcomes in pulmonary arterial hypertension trials. Chest, 149(5), 1234–1244. 10.1016/j.chest.2015.11.008 26836910 PMC4944788

[phy270935-bib-0043] Rosenkranz, S. , Howard, L. S. , Gomberg‐Maitland, M. , & Hoeper, M. M. (2020). Systemic consequences of pulmonary hypertension and right‐sided heart failure. Circulation, 141(8), 678–693. 10.1161/CIRCULATIONAHA.116.022362 32091921

[phy270935-bib-0044] Rothwell, P. M. , & Bhatia, M. (2007). Reporting of Observational Studies (pp. 783–784). British Medical Journal Publishing Group. 10.1136/bmj.39351.581366.BE PMC2034700 17947746

[phy270935-bib-0045] Saunders, L. C. , Johns, C. S. , Stewart, N. J. , Oram, C. J. E. , Capener, D. A. , Puntmann, V. O. , Elliot, C. A. , Condliffe, R. C. , Kiely, D. G. , Graves, M. J. , Wild, J. M. , & Swift, A. J. (2018). Diagnostic and prognostic significance of cardiovascular magnetic resonance native myocardial T1 mapping in patients with pulmonary hypertension. Journal of Cardiovascular Magnetic Resonance, 20(1), 78. 10.1186/s12968-018-0501-8 30501639 PMC6276188

[phy270935-bib-0046] Schulz‐Menger, J. , Bluemke, D. A. , Bremerich, J. , Flamm, S. D. , Fogel, M. A. , Friedrich, M. G. , Kim, R. J. , von Knobelsdorff‐Brenkenhoff, F. , Kramer, C. M. , & Pennell, D. J. (2020). Standardized image interpretation and post‐processing in cardiovascular magnetic resonance‐2020 update: Society for cardiovascular magnetic resonance (SCMR): board of trustees task force on standardized post‐processing. Journal of Cardiovascular Magnetic Resonance, 22(1), 19. 32160925 10.1186/s12968-020-00610-6 PMC7066763

[phy270935-bib-0047] Seemann, F. , Arvidsson, P. , Nordlund, D. , Kopic, S. , Carlsson, M. , Arheden, H. , & Heiberg, E. (2019a). Noninvasive quantification of pressure‐volume loops from brachial pressure and cardiovascular magnetic resonance. Circulation. Cardiovascular Imaging, 12(1), e008493. 10.1161/circimaging.118.008493 30630347

[phy270935-bib-0048] Seemann, F. , Heiberg, E. , Bruce, C. G. , Khan, J. M. , Potersnak, A. , Ramasawmy, R. , Carlsson, M. , Arheden, H. , Lederman, R. J. , & Campbell‐Washburn, A. E. (2024). Non‐invasive pressure–volume loops using the elastance model and CMR: A porcine validation at transient pre‐loads. European Heart Journal ‐ Imaging Methods and Practice, 2(1), qyae016. 38645798 10.1093/ehjimp/qyae016 PMC11026081

[phy270935-bib-0049] Sjögren, H. , Kjellström, B. , Bredfelt, A. , Steding‐Ehrenborg, K. , Rådegran, G. , Hesselstrand, R. , Arheden, H. , & Ostenfeld, E. (2021). Underfilling decreases left ventricular function in pulmonary arterial hypertension. The International Journal of Cardiovascular Imaging, 37(5), 1745–1755. 33502652 10.1007/s10554-020-02143-6 PMC8105202

[phy270935-bib-0050] Sunagawa, K. , Maughan, W. L. , & Sagawa, K. (1985). Optimal arterial resistance for the maximal stroke work studied in isolated canine left ventricle. Circulation Research, 56(4), 586–595. 3978773 10.1161/01.res.56.4.586

[phy270935-bib-0051] Swift, A. J. , Capener, D. , Johns, C. , Hamilton, N. , Rothman, A. , Elliot, C. , Condliffe, R. , Charalampopoulos, A. , Rajaram, S. , Lawrie, A. , Campbell, M. J. , Wild, J. M. , & Kiely, D. G. (2017). Magnetic resonance imaging in the prognostic evaluation of patients with pulmonary arterial hypertension. American Journal of Respiratory and Critical Care Medicine, 196(2), 228–239. 10.1164/rccm.201611-2365OC 28328237 PMC5519970

[phy270935-bib-0052] Swift, A. J. , Rajaram, S. , Campbell, M. J. , Hurdman, J. , Thomas, S. , Capener, D. , Elliot, C. , Condliffe, R. , Wild, J. M. , & Kiely, D. G. (2014). Prognostic value of cardiovascular magnetic resonance imaging measurements corrected for age and sex in idiopathic pulmonary arterial hypertension. Circulation. Cardiovascular Imaging, 7(1), 100–106. 10.1161/CIRCIMAGING.113.000338 24275955

[phy270935-bib-0053] van Wolferen, S. A. , Marcus, J. T. , Boonstra, A. , Marques, K. M. , Bronzwaer, J. G. , Spreeuwenberg, M. D. , Postmus, P. E. , & Vonk‐Noordegraaf, A. (2007). Prognostic value of right ventricular mass, volume, and function in idiopathic pulmonary arterial hypertension. European Heart Journal, 28(10), 1250–1257. 10.1093/eurheartj/ehl477 17242010

[phy270935-bib-0054] Venkateshvaran, A. , Bohlin, J. , Kjellström, B. , Bergström, E. , Nelsson, A. , Werther Evaldsson, A. , Rådegran, G. , Arheden, H. , & Ostenfeld, E. (2024). Left ventricular dysfunction in pulmonary arterial hypertension is attributed to underfilling rather than intrinsic myocardial disease: A CMR 2D phase contrast study. Scientific Reports, 14(1), 17280. 39068288 10.1038/s41598-024-68254-5 PMC11283488

[phy270935-bib-0055] Westerhof, N. , Stergiopulos, N. , & Noble, M. I. (2005). Snapshots of hemodynamics: An aid for clinical research and graduate education. Springer.

