# Supplementary material for: CMR‐derived left ventricular pressure‐volume loops enhance individualized assessment of disease severity and prognosis in pulmonary arterial hypertension in adults
Source: Physiol Rep. 2026 May 31;14(11):e70935. doi: 10.14814/phy2.70935 (PMC13239423; doi:10.14814/phy2.70935)
Supplement: Supplementary file 1 — Figure S1. The graphs show individual changes in SW (above) and MEP (below) from baseline to follow‐up scan in survivors (on the left) and non‐survivors (on the right). Each line represents a single patient and connects the two single values measured in the same individual. Figure S2. The graphs show individual changes in SW (above) and MEP (below) from baseline to follow‐up scan in good‐responders to medical treatment (on the left) and poor‐responders to medical treatment (on the right). Each line represents a single patient and connects the two single values measured in the same individual. Figure S3. The image shows two examples of how MR‐based, non‐invasive PV‐loops changed from baseline to follow‐up in a patient who responded well to PAH‐specific medical treatment (above) and in a patient who did not respond to medical treatment (below). The treatment non‐responder shown here underwent lung transplantation three months after follow‐up. [file PHY2-14-e70935-s002.pdf]

Figure 1

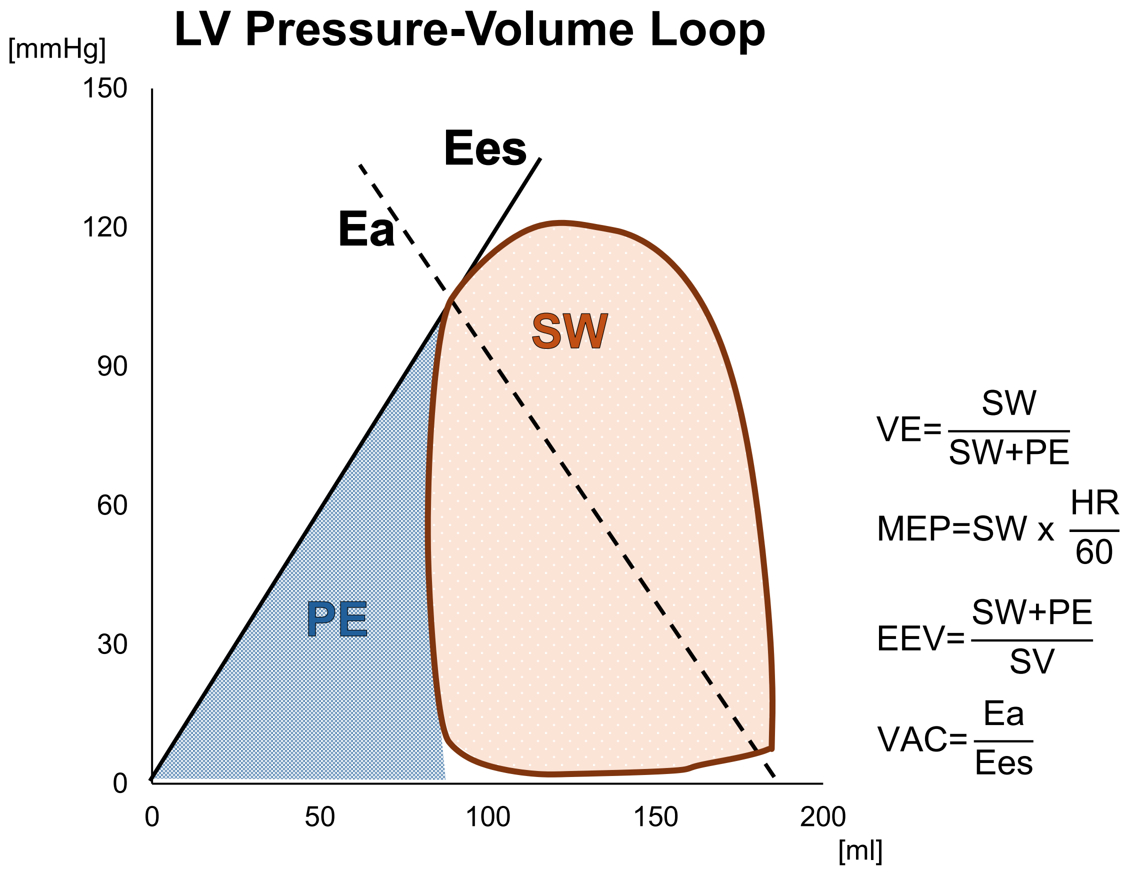

$$VE = \frac{SW}{SW+PE}$$

$$MEP = SW \times \frac{HR}{60}$$

$$EEV = \frac{SW+PE}{SV}$$

$$VAC = \frac{Ea}{Ees}$$

Figure 1: Example of CMR-based, non-invasive PV-loop from a healthy control. The area within the PV-loop (orange) represents stroke work (SW), the blue area represents the potential energy (PE), the solid straight line represents the end-systolic elastance (Ees), used as estimation of LV contractility, and the dashed straight line represents the arterial elastance (Ea), as an estimation of the ventricular afterload.

On the right side, the formulas used to calculate ventricular efficiency (VE), mean external power (MEP), energy per ejected volume (EEV), and ventriculo-arterial coupling (VAC). HR: heart rate; SV: stroke volume.

Figure 2

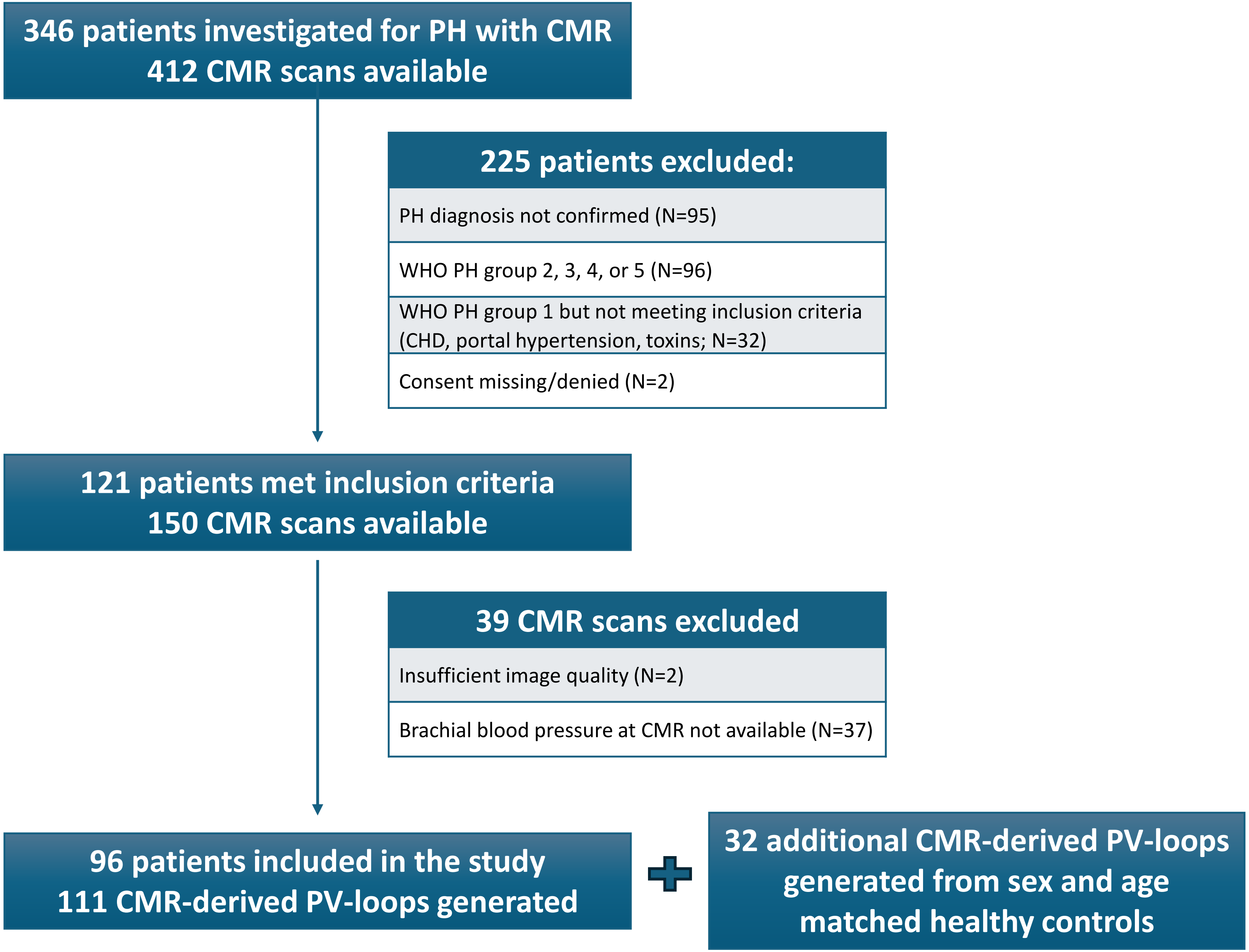

Figure 2: Flow-chart describing selection process used to include patients and healthy controls and to select the CMR scans for the present study.

Figure 3

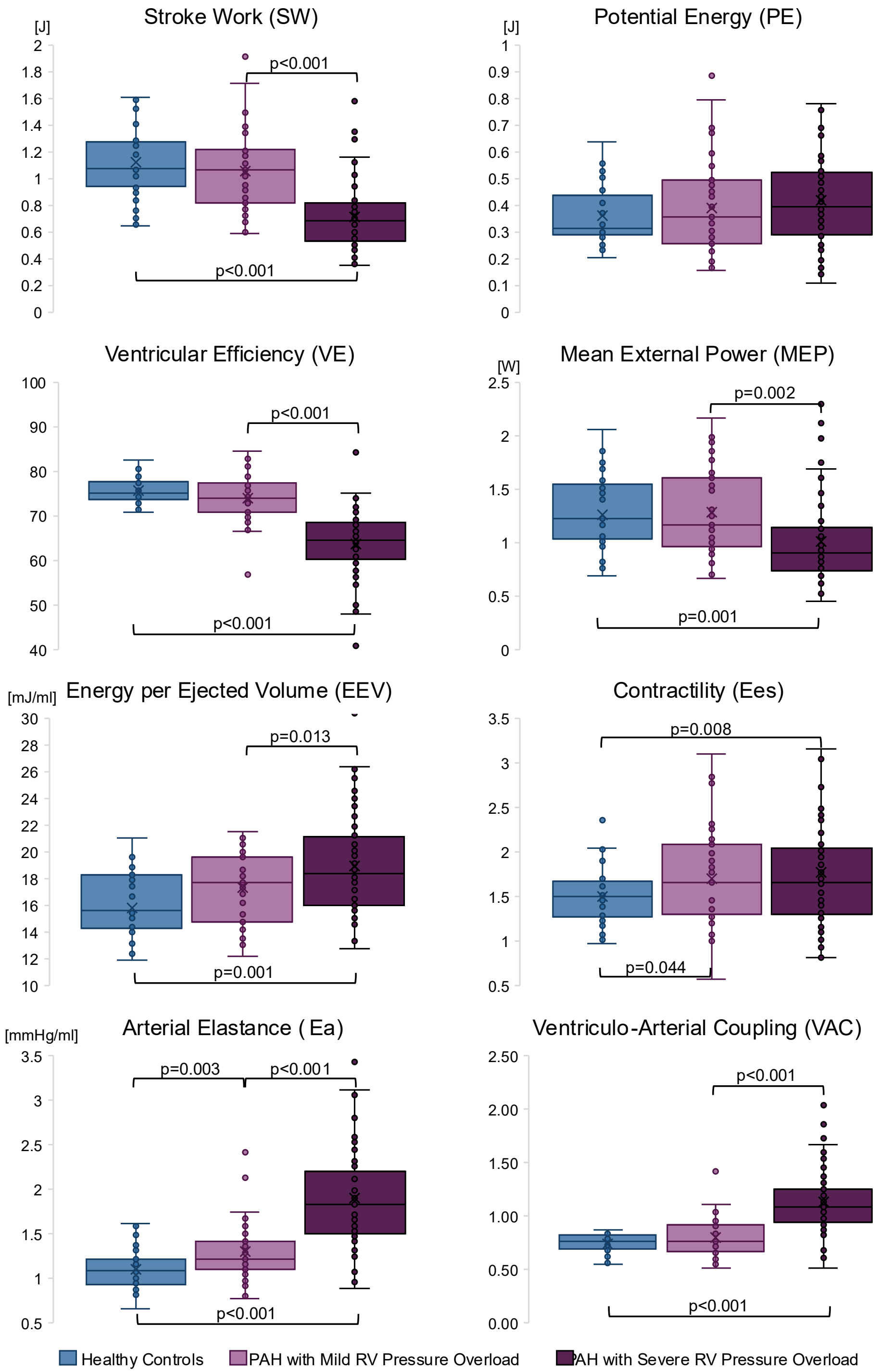

Figure 3: Comparisons of PV-loop derived hemodynamic indices between healthy controls and patients with PAH patients. Patients are presented in according to the degree of RV pressure overload (mild and severe). Reported p values are obtained from unpaired Student's t tests between the groups.

Figure 4

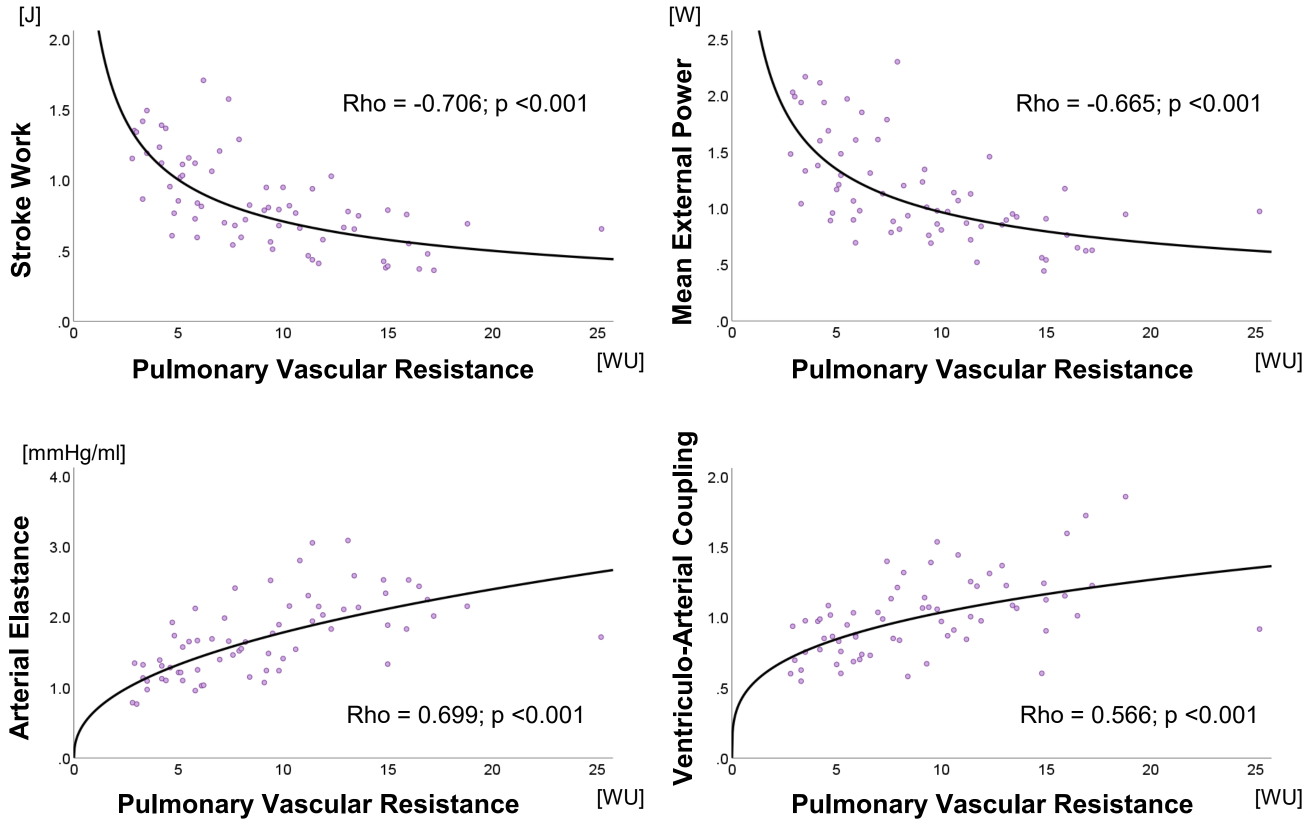

Figure 4: Correlations between pulmonary vascular resistance and stroke work (SW, top left), mean external power (MEP, top right), arterial elastance (Ea, bottom left), and ventriculo-arterial coupling (VAC, bottom right). n=67.

Figure 5

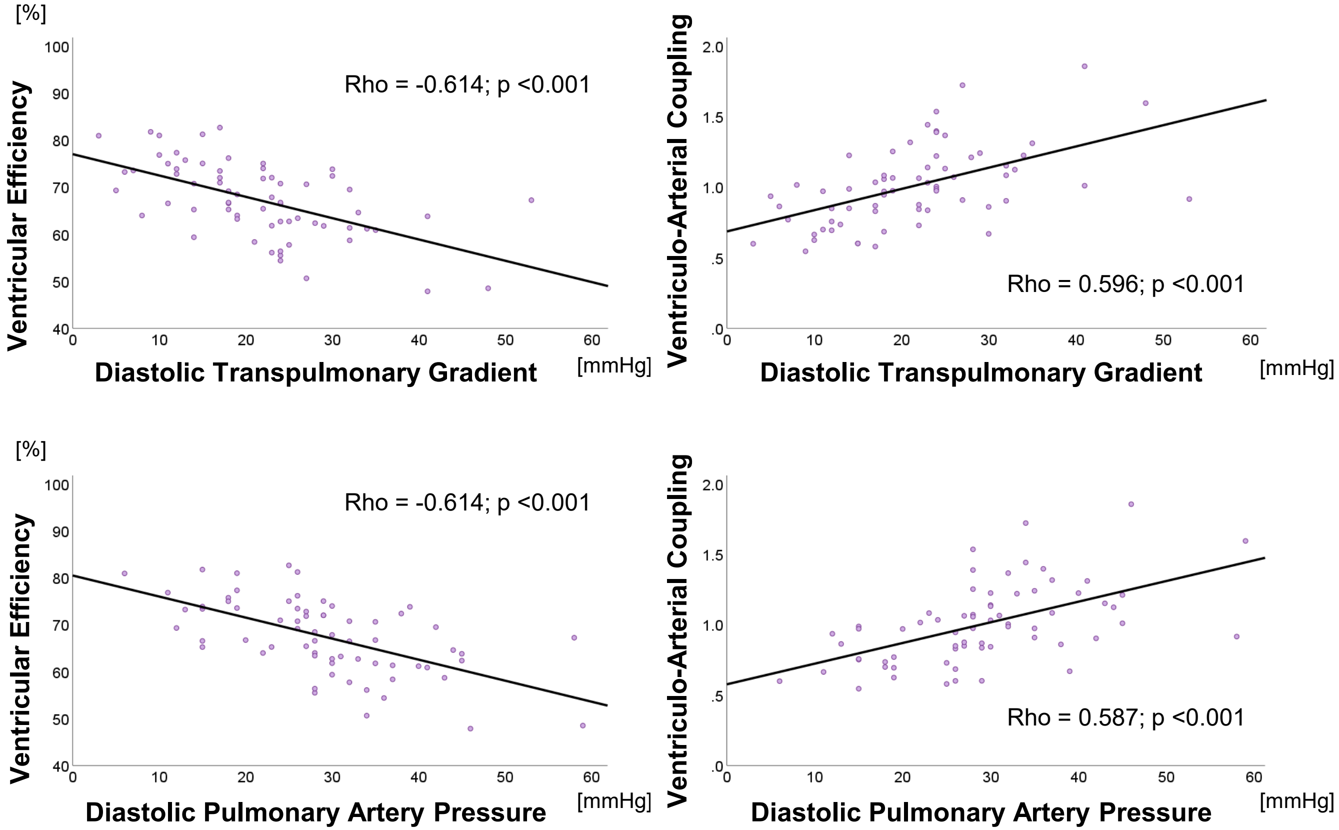

Figure 5: Correlations of diastolic transpulmonary gradient (top row), and diastolic pulmonary arterial pressure (bottom row) versus ventricular efficiency (VE, left) and ventriculo-arterial coupling (VAC, right). n=67.

Figure 6

a)

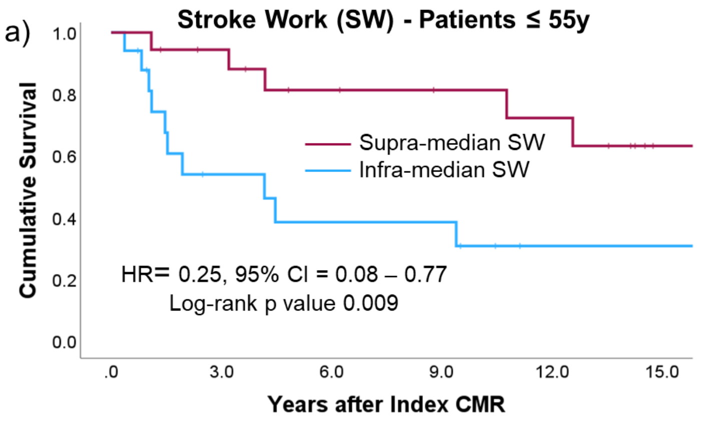

Patients ≤ 55 years		0y	3y	6y	9y	12y	15y
**Infra-median SW**	**N at risk**	17	7	5	5	1	1
	**N of events**	7	2	0	1	0	0
**Supra-median SW**	**N at risk**	18	15	11	9	8	2
	**N of events**	1	2	0	1	1	0

b)

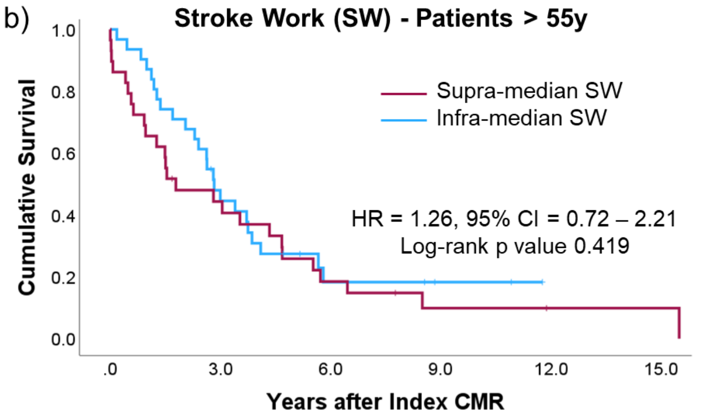

Patients > 55 years		0y	3y	6y	9y	12y	15y
**Infra-median SW**	**N at risk**	31	13	4	2	-	-
	**N of events**	17	7	0	0	-	-
**Supra-median SW**	**N at risk**	29	12	5	2	1	1
	**N of events**	16	7	2	0	0	1

Figure 6: Kaplan-Meier curves and life tables for stroke work: panel a) in the subset of patients younger than 55 y at index CMR; panel b) in the subset of patients older than 55 y at index CMR.

Figure 7

a)

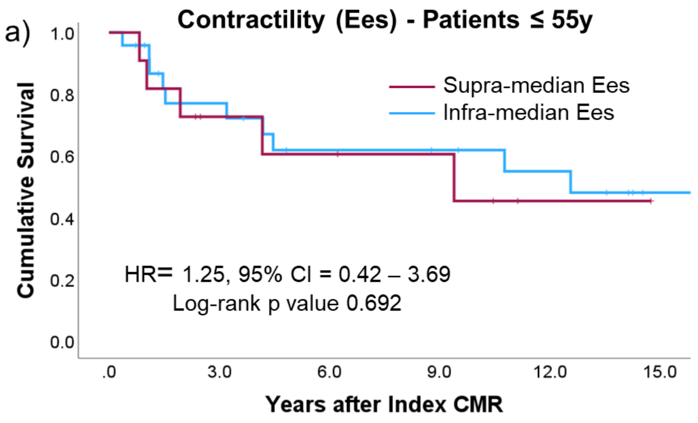

Patients ≤ 55 years		0y	3y	6y	9y	12y	15y
Infra-median Ees	N at risk	24	16	11	10	8	3
	N of events	5	3	0	1	1	0
Supra-median Ees	N at risk	11	6	5	4	1	-
	N of events	3	1	0	1	0	-

b)

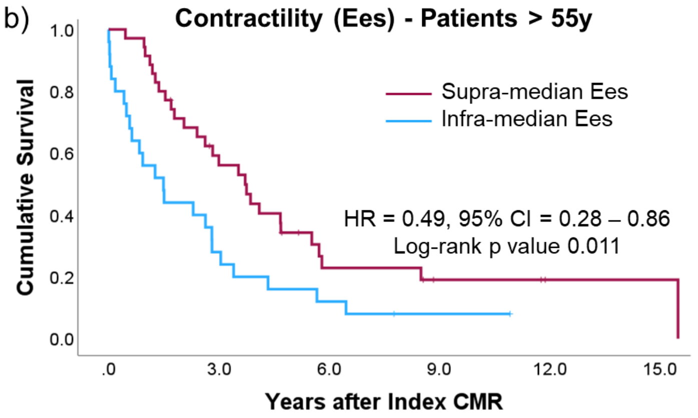

Patients > 55 years		0y	3y	6y	9y	12y	15y
Infra-median SW	N at risk	25	7	3	1	-	-
	N of events	18	4	1	0	-	-
Supra-median SW	N at risk	35	18	6	3	1	1
	N of events	15	10	1	0	0	1

Figure 7: Kaplan-Meier curves and life tables for contractility: panel a) in the subset of patients younger than 55 y at index CMR; panel b) in the subset of patients older than 55 y at index CMR.

Supplementary Figure 1

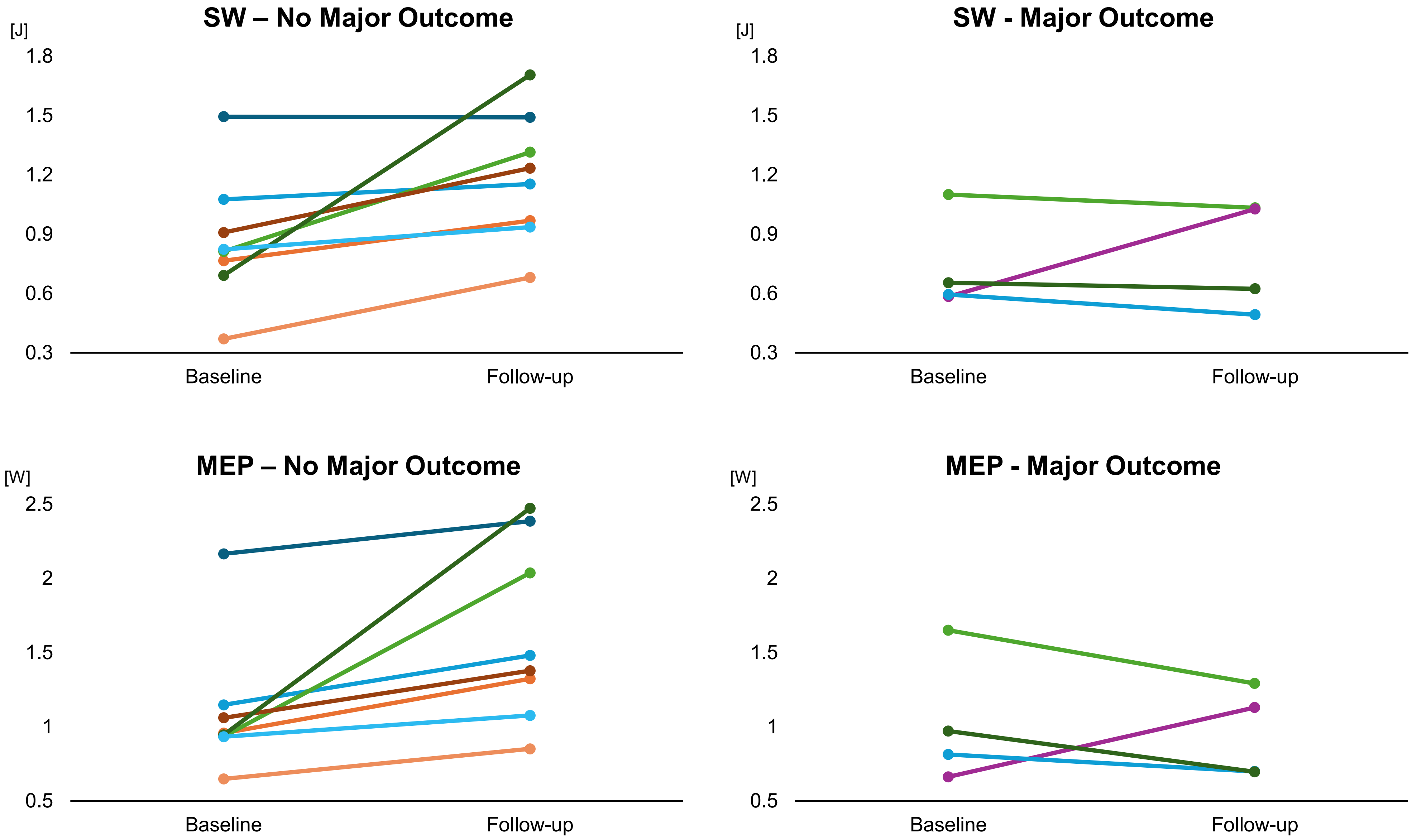

Supplementary figure 1: The graphs show individual changes in SW (above) and MEP (below) from baseline to follow-up scan in survivors (on the left) and non-survivors (on the right). Each line represents a single patient and connects the two single values measured in the same individual.

Supplementary Figure 2

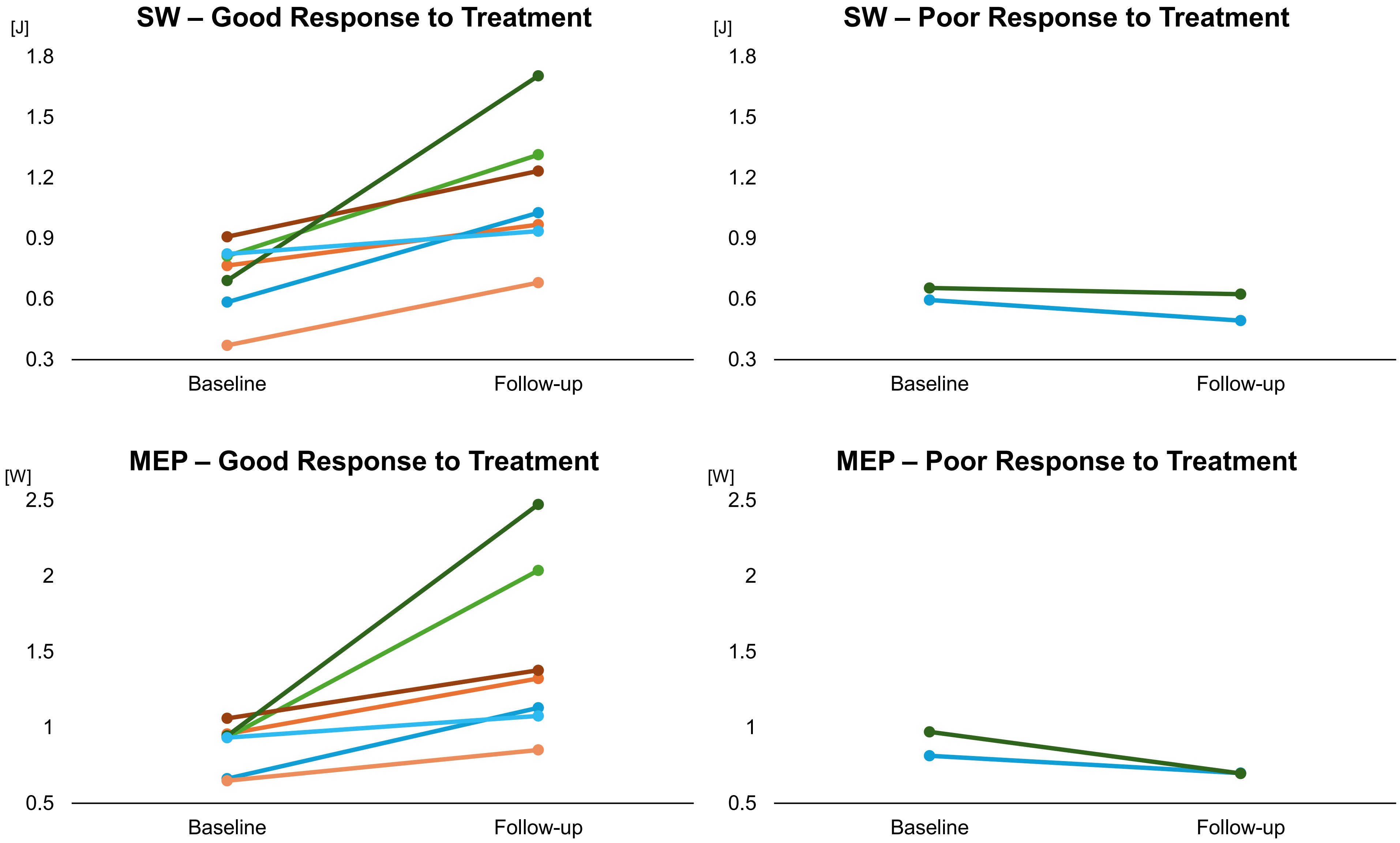

Supplementary figure S2: The graphs show individual changes in SW (above) and MEP (below) from baseline to follow-up scan in good-responders to medical treatment (on the left) and poor-responders to medical treatment (on the right). Each line represents a single patient and connects the two single values measured in the same individual.

Supplementary Figure 3

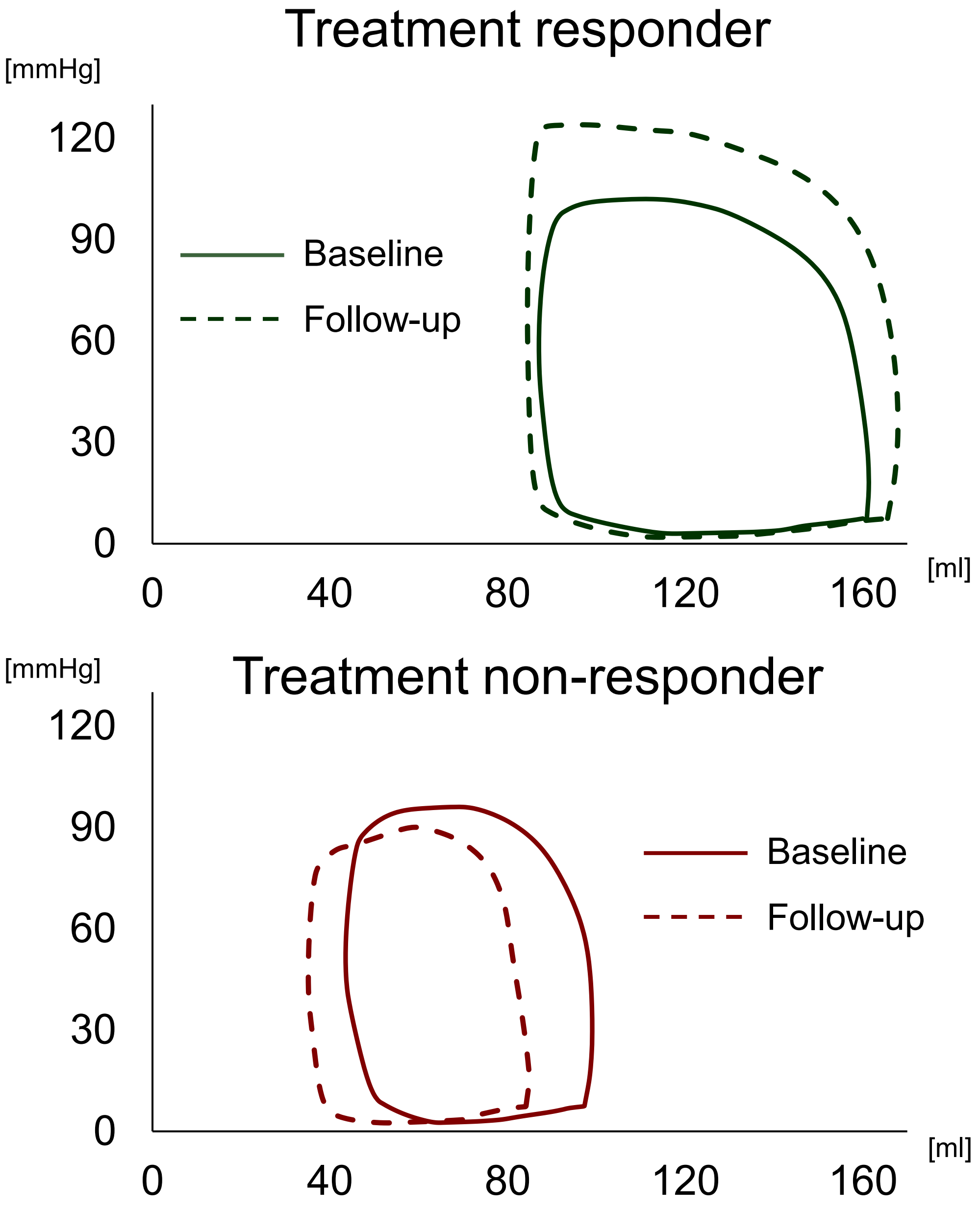

Supplementary figure S3: The image shows two examples of how MR-based, non-invasive PV-loops changed from baseline to follow-up in a patient who responded well to PAH-specific medical treatment (above) and in a patient who did not respond to medical treatment (below). The treatment non-responder shown here underwent lung transplantation three months after follow-up.
