# Supplementary material for: CMR‐derived left ventricular pressure‐volume loops enhance individualized assessment of disease severity and prognosis in pulmonary arterial hypertension in adults
Source: Physiol Rep. 2026 May 31;14(11):e70935. doi: 10.14814/phy2.70935 (PMC13239423; doi:10.14814/phy2.70935)
Supplement: Supplementary file 2 — Table S1. Age‐Stratified Baseline Participant Characteristics. Table S2. Age‐Stratified Baseline CMR and PV‐loop findings. Table S3. Univariate Cox regression analysis. Table S4. Repeat scans patient characteristics. Table S5. Repeat scans patient CMR and PV‐loop findings. Table S6. PV‐loop indices in repeat scans in non‐survivors and survivors. [file PHY2-14-e70935-s001.docx]

**SUPPLEMENTARY TABLE S1**

| Supplementary Table 1: Age-Stratified Baseline Participant Characteristics | | | |
| --- | --- | --- | --- |
|  | **Patients ≤ 55 years**  **(n = 35)** | **Patients > 55 years**  **(n = 61)** | ***P* value** |
| Clinical | | | |
| Age [y] | 33 [25 - 45.5] | 71 [65 – 74] | - |
| Female | 26 (74%) | 41 (67%) | 0.499 |
| BMI [kg/m^2^] | 25.4 ± 6.2 | 25.5 ± 3.8 | 0.899 |
| BSA [m^2^] | 1.81 ± 0.27 | 1.81 ± 0.21 | 0.975 |
| PAH diagnosis group  - IPAH/HPAH  - APAH | 25 (71%)  10 (29%) | 25 (41%)  36 (59%) | **0.004** |
| Time since PAH diagnosis  - Prevalent case  - Incident case | 11 (31%)  24 (69%) | 19 (31%)  42 (69%) | 0.576 |
| PAH treatment at CMR  - No treatment  - Monotherapy  - Dual therapy  - Triple therapy | 22 (63%)  7 (20%)  1 (3%)  5 (14%) | 31 (51%)  14 (23%)  14 (23%)  2 (3%) | **0.018** |
| Comorbidities | | | |
| Diabetes | 5 (14%) | 9 (15%) | 0.950 |
| COPD | 1 (3%) | 16 (26%) | **0.004** |
| Raynaud’s disease | 11 (31%) | 25 (41%) | 0.352 |
| Ischemic heart disease | 3 (9%) | 9 (15%) | 0.378 |
| Arterial hypertension | 9 (26%) | 24 (39%) | 0.275 |
| Thyroid disease | 4 (11%) | 11 (18%) | 0.391 |
| Atrial fibrillation | 3 (9%) | 10 (16%) | 0.549 |
| Stroke | 0 | 5 (8%) | 0.286 |
| Dyslipidemia | 2 (6%) | 7 (12%) | 0.126 |
| Right Heart Catheterization | | | |
| mPAP [mmHg] | 53 ± 14 ^a^ | 44 ± 12 ^b^ | **0.012** |
| dPAP [mmHg] | 35 ± 11 ^a^ | 27 ± 8 ^b^ | **0.003** |
| sPAP [mmHg] | 80 ± 19 ^a^ | 71 ± 20 ^b^ | 0.094 |
| PAWP [mmHg] | 8 ± 4 ^a^ | 8 ± 4 ^b^ | 0.976 |
| TPG [mmHg] | 44 ± 14 ^a^ | 36 ± 11 ^b^ | **0.011** |
| DPG [mmHg] | 27 ± 11 ^a^ | 19 ± 8 ^b^ | **0.003** |
| PVR [WU] | 11.4 ± 5.4 ^a^ | 8.3 ± 3.7 ^b^ | **0.007** |
| SVR [WU] | 23.0 ± 8.9 ^a^ | 20.3 ± 7.4 ^b^ | 0.165 |
| mRAP [mmHg] | 8 ± 6 ^a^ | 8 ± 5 ^b^ | 0.942 |
| RVEDP [mmHg] | 12 ± 8 ^a^ | 12 ± 6 ^b^ | 0.773 |
| RVSP [mmHg] | 76 ± 22 ^a^ | 70 ± 20 ^b^ | 0.266 |

Supplementary table S1: Baseline characteristics of PAH patients divided into age category subsets. Data expressed as mean ± SD or median [IQR]. Student’s t-test, and χ² test were used to test for differences as appropriate; p values <0.05 are highlighted in bold.

N numbers diverging from the ones reported on the top of the table are indicated with ^a^ for n= 23, and ^b^ for n= 40.

APAH: associated pulmonary arterial hypertension; BMI: body mass index; BSA: body surface area; COPD: chronic obstructive pulmonary disease; dPAP: diastolic pulmonary arterial pressure; DPG: diastolic transpulmonary gradient; IPAH/HPAH: idiopathic or hereditary pulmonary arterial hypertension; mPAP: mean pulmonary arterial pressure; mRAP: mean right atrial pressure; PAWP: pulmonary arterial wedge pressure; PVR: pulmonary vascular resistance; RVEDP: right ventricular end-diastolic pressure; RVSP: right ventricular systolic pressure; sPAP: systolic pulmonary arterial pressure; SVR: systemic vascular resistance; TPG: transpulmonary gradient.

**SUPPLEMENTARY TABLE S2**

| Supplementary Table 2: Age-Stratified Baseline CMR and PV-loop findings | | | |
| --- | --- | --- | --- |
|  | **Patients ≤ 55 years**  **(n = 35)** | **Patients > 55 years**  **(n = 61)** | ***P* value** |
| Clinical | | | |
| Non-invasive blood pressure  - Systolic [mmHg]  - Diastolic [mmHg] | 118 ± 16  78 ± 13 | 125 ± 18  75 ± 12 | 0.059  0.202 |
| Heart rate [bpm] | 77 ± 15 | 80 ± 13 | 0.259 |
| CMR | | | |
| LVEDV [ml] | 132 ± 39 | 129 ± 42 | 0.754 |
| LVEDVI [ml/m^2^] | 73 ±19 | 71 ± 20 | 0.606 |
| LVESV [ml] | 65 ± 20 | 60 ± 25 | 0.241 |
| LVESVI [ml/m^2^] | 36 ± 10 | 32 ± 12 | 0.138 |
| LVSV [ml] | 67 ± 23 | 70 ± 21 | 0.490 |
| LVSVI [ml/m^2^] | 37 ± 12 | 39 ± 10 | 0.539 |
| LVEF [%] | 50 ± 7 | 55 ± 7 | **0.005** |
| LVM [g] | 63 ± 17 | 68 ± 25 | 0.290 |
| LVMI [g/m^2^] | 35 ± 6 | 37 ± 11 | 0.221 |
| CO [l/min] | 5.0 ± 1.7 | 5.5 ± 1.7 | 0.160 |
| CI [l/min/ m^2^] | 2.8 ± 0.9 | 3.0 ± 0.8 | 0.160 |
| RVEDV [ml] | 260 ± 81 | 225 ± 72 | **0.030** |
| RVEDVI [ml/m^2^] | 144 ± 39 | 123 ± 33 | **0.007** |
| RVESV [ml] | 184 ± 76 | 147 ± 67 | **0.014** |
| RVESVI [ml/m^2^] | 101 ± 38 | 80 ± 33 | **0.005** |
| RVSV [ml] | 76 ± 20 | 78 ± 21 | 0.604 |
| RVSVI [ml/m^2^] | 42 ± 11 | 43 ± 10 | 0.716 |
| RVEF [%] | 31 ± 10 | 37 ± 12 | **0.010** |
| RV Pressure Overload Score  - Mild  - Severe | 10 (28.6%)  25 (71.4%) | 29 (47.5%)  32 (52.5%) | 0.057 |
| PV-loop Indices | | | |
| SW [J] | 0.8 ± 0.3 | 0.9 ± 0.3 | 0.256 |
| PE [J] | 0.4 ± 0.2 | 0.4 ±0.2 | 0.495 |
| VE | 65 ± 9 | 70 ± 8 | **0.011** |
| MEP [W] | 1.0 ± 0.5 | 1.2 ± 0.4 | 0.117 |
| EEV [mJ/ml] | 19 ± 4 | 18 ± 3 | 0.642 |
| Ees [mmHg/ml] | 1.6 ± 0.4 | 1.9 ± 0.7 | **0.019** |
| Ea [mmHg/ml] | 1.7 ± 0.6 | 1.6 ± 0.6 | 0.619 |
| VAC | 1.1 ± 0.3 | 0.9 ± 0.3 | **0.005** |

Supplementary table S2: Findings from CMR and PV-loop analysis from PAH patients divided into age category subsets. Data expressed as mean ± SD. Student’s t-test, and χ² test were used to test for differences as appropriate. p values <0.05 are highlighted in bold.

Abbreviations CI: cardiac index; CO: cardiac output; Ea: left ventricular arterial elastance; Ees: left ventricular end-systolic elastance; EEV: left ventricular energy per ejected volume; LVEDV: left ventricular end-diastolic volume; LVEDVI: BSA-indexed left ventricular end-diastolic volume; LVEF: left ventricular ejection fraction; LVESV: left ventricular end-systolic volume; LVESVI: BSA-indexed left ventricular end-systolic volume; LVM: left ventricular mass; LVMI: BSA-indexed left ventricular mass; LVSV: left ventricular stroke volume; LVSVI: BSA-indexed left ventricular stroke volume MEP: left ventricular mean external power; PE: left ventricular potential energy; RVEDV: right ventricular end-diastolic volume; RVEDVI: BSA-indexed right ventricular end-diastolic volume; RVEF: right ventricular ejection fraction; RVESV: right ventricular end-systolic volume; RVESVI: BSA-indexed right ventricular end-systolic volume; RVSV: right ventricular stroke volume; RVSVI: BSA-indexed right ventricular stroke volume;SW: left ventricular stroke work; VAC: left ventricular ventriculo-arterial coupling; VE: left ventricular efficiency.

**SUPPLEMENTARY TABLE S3**

| Supplementary Table S3: Univariate Cox regression analysis | | | | | | |
| --- | --- | --- | --- | --- | --- | --- |
|  | **Whole patient cohort**  (n=95, Outcome = 65) | | **Patients ≤ 55 years**  **(**n=35, Outcome = 15) | | **Patients > 55 years**  n=60, Outcome = 50 | |
|  | **HR** | **P value** | **HR** | ***P* value** | **HR** | ***P* value** |
| Supra-median LVSV | 0.93 | 0.759 | 0.57 | 0.303 | 1.07 | 0.824 |
| Supra-median LVSVI | 1.17 | 0.543 | 0.75 | 0.596 | 1.35 | 0.293 |
| Supra-median SBP | 0.96 | 0.859 | 0.44 | 0.163 | 1.16 | 0.606 |
| Supra-median MBP | 0.77 | 0.309 | 0.54 | 0.274 | 0.82 | 0.459 |
| Supra-median DBP | 0.83 | 0.472 | 0.66 | 0.442 | 0.95 | 0.853 |

Supplementary Table S3: Univariate Cox regression analysis of patients with pulmonary arterial hypertension as whole cohort and clustered in age categories of patients ≤ 55y and patients > 55y at index CMR scan**.** Data expressed as hazard ratios (HR) with their respective p values.

DBP: diastolic systemic arterial pressure; LVSV: left ventricular stroke volume; LVSVI: BSA-indexed left ventricular stroke volume; MBP: mean systemic arterial pressure; SBP: systolic systemic arterial pressure.

**SUPPLEMENTARY TABLE S4**

| Supplementary table S4: Repeat scans patient characteristics | | | |
| --- | --- | --- | --- |
| n=12 | **Baseline** | **Follow-Up** | **P value** |
| Clinical | | | |
| Median age, [y] | 48 [30 – 70] | 48.5 [32 – 71] | - |
| Female Sex | 8 (67%) | - | - |
| Diagnosis  - IPAH  - APAH | 7 (58%)  5 (42%) | 7 (58%)  5 (42%) | - |
| PAH medication at CMR examination  - No treatment  - Monotherapy  - Double therapy  - Triple therapy | 9 (75%)  2 (17%)  1 (8%)  0 | 1 (8%)  4 (33%)  5 (42%)  2 (17%) | **0.008** |
| PAH medication changes between  CMR examinations:  - No changes  - Start of therapy  - Escalation of therapy  - Deescalation of therapy | -  -  -  - | 2 (17%)  8 (67%)  1 (8%)  1 (8%) | - |
| Comorbidities | | | |
| Diabetes | 3 (5%) | 3 (25%) | - |
| COPD | 0 | 0 | - |
| Raynaud’s disease | 6 (50%) | 6 (50%) | - |
| Ischemic heart disease | 1 (8%) | 1 (8%) | - |
| Arterial hypertension | 5 (42%) | 5 (42%) | - |
| Thyroid disease | 0 | 0 | - |
| Atrial fibrillation | 0 | 0 | - |
| Stroke | 0 | 0 | - |
| Dyslipidemia | 2 (17%) | 2 (17%) | - |
| Right Heart Catheterization | | | |
| mPAP [mmHg] | 45 [41 – 65] ^a^ | 36 [28 – 46] ^b^ | 0.063 |
| dPAP [mmHg] | 29 [25 – 46] ^a^ | 26 [14 – 28] ^b^ | **0.028** |
| sPAP [mmHg] | 73 [53 – 101] ^a^ | 56 [43 – 73] ^b^ | 0.116 |
| PAWP [mmHg] | 6 [5 – 8] ^a^ | 8 [6 – 10] ^b^ | 0.090 |
| TPG [mmHg] | 37 [29 – 61] ^a^ | 29 [18 – 37] ^b^ | 0.063 |
| DPG [mmHg] | 23 [17 – 41] ^a^ | 17 [4 – 21] ^b^ | **0.028** |
| PVR [WU] | 8.4 [4.8 – 19.0] ^a^ | 4.6 [3.3 – 7.7] ^b^ | **0.018** |
| RVEDP [mmHg] | 8 [5 – 12] ^a^ | 9 [6 – 11] ^b^ | 0.750 |
| RVSP [mmHg] | 66 [49 – 101] ^a^ | 55 [46 – 72] ^b^ | 0.176 |

Supplementary table S4: Patient characteristics and survival data of from the subset of PAH patients with repeat CMR scans. Data are reported in median and [IQR] or absolute numbers and percentage (%). Wilcoxon signed-rank test was used to assess differences between baseline and follow-up values for continuous variables, χ^2^ square for categorical variables. p values <0.05 are highlighted in bold.

N numbers diverging from the one reported on the top of the table are indicated with ^a^ for n= 7, and ^b^ for n= 10.

APAH: associated pulmonary arterial hypertension; BMI: body mass index; BSA: body surface area; COPD: chronic obstructive pulmonary disease; dPAP: diastolic pulmonary arterial pressure; DPG: diastolic transpulmonary gradient; IPAH/HPAH: idiopathic or hereditary pulmonary arterial hypertension; mPAP: mean pulmonary arterial pressure; PAWP: pulmonary arterial wedge pressure; PVR: pulmonary vascular resistance; RVEDP: right ventricular end-diastolic pressure; RVSP: right ventricular systolic pressure; sPAP: systolic pulmonary arterial pressure; TPG: transpulmonary gradient.

**SUPPLEMENTARY TABLE S5**

| Supplementary table S5: Repeat scans patient CMR and PV-loop findings | | | |
| --- | --- | --- | --- |
| n=12 | **Baseline** | **Follow-Up** | **P value** |
| Clinical | | | |
| Non-invasive blood pressure  - Systolic [mmHg]  - Diastolic [mmHg] | 113 [104 – 128]  73 [70 – 80] | 123 [111 – 133]  76 [64 – 83] | 0.433  0.455 |
| Heart rate [bpm] | 75 [66 – 85] | 75 [75 – 83] | 0.934 |
| CMR | | | |
| LVEDV [ml] | 125 [104 – 147] | 141 [126 – 167] | **0.019** |
| LVEDVI [ml/m2] | 68 [60 – 78] | 82 [70 – 94] | **0.010** |
| LVESV [ml] | 51 [41 – 68] | 60 [50 – 74] | 0.182 |
| LVESVI [ml/m2] | 30 [24 – 36] | 35 [29 – 41] | 0.099 |
| LVSV [ml] | 65 [56 – 81] | 85 [72 – 97] | **0.019** |
| LVSVI [ml/m2] | 36 [31 – 46] | 47 [40 – 52] | **0.010** |
| LVEF [%] | 55 [53 – 58] | 56 [53 – 60] | 0.875 |
| LVM [g] | 63 [46 – 81] | 62 [54 – 79] | 0.433 |
| LVMI [g/m2] | 33 [26 – 42] | 37 [31 – 43] | 0.209 |
| CO [l/min] | 4.9 [4.3 – 5.4] | 6.0 [4.6 – 7.9] | 0.060 |
| CI [l/min/ m2] | 2.9 [2.5 – 3.2] | 3.6 [2.7 – 4.1] | **0.034** |
| RVEDV [ml] | 190 [157 – 224] | 194 [156 – 244] | 0.937 |
| RVEDVI [ml/m2] | 111 [93 – 129] | 118 [95 – 136] | 0.530 |
| RVESV [ml] | 118 [93 – 151] | 105 [74 – 163] | 0.060 |
| RVESVI [ml/m2] | 66 [54 – 86] | 64 [46 – 83] | 0.117 |
| RVSV [ml] | 68 [58 – 85] | 87 [79 – 98] | **0.008** |
| RVSVI [ml/m2] | 40 [32 – 46] | 49 [45 – 55] | **0.008** |
| RVEF [%] | 40 [28 – 46] | 47 [37 – 52] | **0.005** |
| RV Pressure Overload Risk Category  - Mild  - Severe | 6 (50%)  6 (50%) | 5 (42%)  7 (58%) | - |
| PV-loop Indices | | | |
| SW [J] | 0.8 [0.6 – 1.0] | 1.0 [0.9 – 1.3] | **0.028** |
| PE [J] | 0.3 [0.2 – 0.5] | 0.4 [0.3 – 0.5] | 0.136 |
| VE | 72 [67 – 74] | 72 [70 – 76] | 0.583 |
| MEP [W] | 1.0 [0.9 – 1.1] | 1.3 [1.0 – 1.6] | 0.050 |
| EEV [mJ/ml] | 16 [15 – 18] | 16 [15 – 19] | 0.814 |
| Ees [mmHg/ml] | 1.8 [1.2 – 2.0] | 1.5 [1.3 – 1.8] | 0.308 |
| Ea [mmHg/ml] | 1.5 [1.2 – 1.7] | 1.2 [1.2 – 1.4] | 0.136 |
| VAC | 0.9 [0.8 – 1.0] | 0.9 [0.7 – 0.9] | 0.388 |

Supplementary table S5: Findings from CMR and PV-loop analysis from the subset of PAH patients with repeat CMR scans. Data are reported in median and [IQR]. Wilcoxon signed-rank test was used to assess differences between baseline and follow-up values for continuous variables. p values <0.05 are highlighted in bold.

CI: cardiac index; CO: cardiac output; Ea: left ventricular arterial elastance; Ees: left ventricular end-systolic elastance; EEV: left ventricular energy per ejected volume; LVEDV: left ventricular end-diastolic volume; LVEDVI: BSA-indexed left ventricular end-diastolic volume; LVEF: left ventricular ejection fraction; LVESV: left ventricular end-systolic volume; LVESVI: BSA-indexed left ventricular end-systolic volume; LVM: left ventricular mass; LVMI: BSA-indexed left ventricular mass; LVSV: left ventricular stroke volume; LVSVI: BSA-indexed left ventricular stroke volume; MEP: left ventricular mean external power; PE: left ventricular potential energy; RVEDV: right ventricular end-diastolic volume; RVEDVI: BSA-indexed right ventricular end-diastolic volume; RVEF: right ventricular ejection fraction; RVESV: right ventricular end-systolic volume; RVESVI: BSA-indexed right ventricular end-systolic volume; RVSV: right ventricular stroke volume; RVSVI: BSA-indexed right ventricular stroke volume; SW: left ventricular stroke work; VAC: left ventricular ventriculo-arterial coupling; VE: left ventricular efficiency.

**SUPPLEMENTARY TABLE S6**

| Supplementary table S6: PV-loop indices in repeat scans in non-survivors and survivors | | | | | |  |
| --- | --- | --- | --- | --- | --- | --- |
|  | **Non-Survivors (n=4)** | | **Survivors (n=8)** | |  |  |
|  | **Baseline** | **Change (%)** | **Baseline** | **Change (%)** | **P value** | |
| SW [J] | 0.6 [0.6; 0.8] | -5.4 [-8.8; +15.4] | 0.8 [0.8; 1.0] | +31.1 [+12.1; +67.2] | 0.109 | |
| PE [J] | 0.3 [0.2; 0.4] | -9.4 [-31.1; +37.3] | 0.3 [0.3; 0.5] | +35.6 [+10.9; +44.7] | 0.368 | |
| VE | 72 [70; 74] | -0.4 [-4.4; +5.9] | 70 [66; 74] | +1.9 [-4.7; +7.0] | 0.808 | |
| MEP [W] | 0.9 [0.8; 1.1] | -17.9 [-23.4; +7.1] | 1.0 [0.9; 1.1] | +30.6 [+25.6; +58.2] | 0.109 | |
| EEV [mJ/ml] | 16 [14; 18] | -10.1 [-15.1; +3.0] | 17 [15; 18] | +6.3 [-5.6; +19.3] | 0.368 | |
| Ees [mmHg/ml] | 1.9 [1.8; 2.0] | -0.5 [-17.5; +11.3] | 1.5 [1.1; 2.0] | +0.6 [-29.2; +16.3] | 0.683 | |
| Ea [mmHg/ml] | 1.5 [1.5; 1.6] | -12.4 [-25.2; +1.4] | 1.2 [1.1; 1.8] | -14.5 [-35.8; +12.3] | 1.000 | |
| VAC | 0.9 [0.8; 0.9] | +2.3 [-17.9; +18.0] | 0.9 [0.8; 1.0] | -5.1 [-10.7; +2.0] | 0.808 | |

Supplementary table S6: PV-loop derived indices at baseline and the relative change (in %) at follow-up in percentage from baseline, clustered in non-survivors and survivors. Data reported in median value [IQR]. Comparison between changes in survivors vs. changes in non-survivors performed with Mann–Whitney U Test.

Ea: left ventricular arterial elastance; Ees: left ventricular end-systolic elastance; EEV: left ventricular energy per ejected volume; MEP: left ventricular mean external power; PE: left ventricular potential energy; SW: left ventricular stroke work; VAC: left ventricular ventriculo-arterial coupling; VE: left ventricular efficiency.

The last column on the right reports Mann-Whitney U test p values for the difference between baseline values in non-survivors and survivors.
